# Cytoplasmic CPSF6 Regulates HIV-1 Capsid Trafficking and Infection in a Cyclophilin A-Dependent Manner

**DOI:** 10.1128/mBio.03142-20

**Published:** 2021-03-23

**Authors:** Zhou Zhong, Jiying Ning, Emerson A. Boggs, Sooin Jang, Callen Wallace, Cheryl Telmer, Marcel P. Bruchez, Jinwoo Ahn, Alan N. Engelman, Peijun Zhang, Simon C. Watkins, Zandrea Ambrose

**Affiliations:** a Department of Microbiology and Molecular Genetics, University of Pittsburgh School of Medicine, Pittsburgh, Pennsylvania, USA; b Department of Structural Biology, University of Pittsburgh School of Medicine, Pittsburgh, Pennsylvania, USA; c Department of Cell Biology, University of Pittsburgh School of Medicine, Pittsburgh, Pennsylvania, USA; d Pittsburgh Center for HIV Protein Interactions, University of Pittsburgh School of Medicine, Pittsburgh, Pennsylvania, USA; e Department of Cancer Immunology and Virology, Dana-Farber Cancer Institute, Boston, Massachusetts, USA; f Department of Medicine, Harvard Medical School, Boston, Massachusetts, USA; g Department of Biological Sciences, Carnegie Mellon University, Pittsburgh, Pennsylvania, USA; h Division of Structural Biology, Wellcome Trust Centre for Human Genetics, University of Oxford, Oxford, England; i Electron Bio-Imaging Centre, Diamond Light Source, Harwell Science and Innovation Campus, Didcot, United Kingdom; Ohio State University; University of Pittsburgh School of Medicine

**Keywords:** CPSF6, capsid, cyclophilin A, human immunodeficiency virus, live-cell imaging, microtubule

## Abstract

Human immunodeficiency virus type 1 (HIV-1) capsid binds host proteins during infection, including cleavage and polyadenylation specificity factor 6 (CPSF6) and cyclophilin A (CypA). We observe that HIV-1 infection induces higher-order CPSF6 formation, and capsid-CPSF6 complexes cotraffic on microtubules. CPSF6-capsid complex trafficking is impacted by capsid alterations that reduce CPSF6 binding or by excess cytoplasmic CPSF6 expression, both of which are associated with decreased HIV-1 infection. Higher-order CPSF6 complexes bind and disrupt HIV-1 capsid assemblies *in vitro*. Disruption of HIV-1 capsid binding to CypA leads to increased CPSF6 binding and altered capsid trafficking, resulting in reduced infectivity. Our data reveal an interplay between CPSF6 and CypA that is important for cytoplasmic capsid trafficking and HIV-1 infection. We propose that CypA prevents HIV-1 capsid from prematurely engaging cytoplasmic CPSF6 and that differences in CypA cellular localization and innate immunity may explain variations in HIV-1 capsid trafficking and uncoating in CD4^+^ T cells and macrophages.

## Abstract (precis)

HIV is the causative agent of AIDS, which has no cure. The protein shell that encases the viral genome, the capsid, is critical for HIV replication in cells at multiple steps.

## INTRODUCTION

Human immunodeficiency virus type 1 (HIV-1) capsid is a unique structure that assembles after viral proteolytic cleavage of the Gag and Gag-Pol polyproteins during or after virus budding and release from cells. Multiple HIV-1 capsid proteins (CAs) form a conical-shaped core consisting of approximately 200 hexamers and 12 pentamers, which encapsulates two copies of the RNA genome (1, 2). An important yet understudied aspect of HIV-1 infection, which is referred to as uncoating, describes the process of capsid dissociation that occurs during reverse transcription and before viral DNA integration into host chromatin (3, 4). HIV-1 capsid uncoating is dependent on microtubule trafficking (5–7) and may occur in a multistep process (8). HIV-1 infection requires active transport of the viral reverse transcription complex/preintegration complex (RTC/PIC) through the cellular nuclear pore complex (NPC) (9), and the majority of capsid is likely uncoated at the NPC and/or within the nucleus (10–14). Single mutations in CA can greatly impact the stability of HIV-1 capsid, altering its uncoating and affecting virus infectivity (15, 16).

HIV-1 capsid has been shown to bind several host proteins during infection (17). Two examples are cyclophilin A (CypA) (18, 19), which is relatively abundant in cells (20), and cleavage and polyadenylation specificity factor 6 (CPSF6) (21). Disruption of HIV-1 capsid binding to CypA can occur via amino acid substitution, such as G89V and P90A, in the loop between helices 4 and 5 in CA (22, 23) or by treatment with small-molecule inhibitors, such as cyclosporine A (CsA) (18). The inability to bind to CypA in target cells can affect the infectivity of the invading HIV-1 particle (24). CypA dependence is cell type specific and has been shown to affect multiple steps in the virus life cycle, including capsid uncoating, reverse transcription, nuclear import, integration, and evasion of TRIM5α restriction (25–30).

CPSF6 is an arginine/serine (RS) domain-containing protein expressed predominantly in the cell nucleus and is involved in splicing and polyadenylation of host RNAs (31). CPSF6 binds to HIV-1 capsid at an interface between two CA monomers defined by helices 3, 4, and 5 (21, 32). The beta-karyopherin transportin 3 (TNPO3) is the key mediator of RS domain protein nucleocytoplasmic transport in cells, and TNPO3 directly binds the C-terminal RS domain in CPSF6 to affect its nuclear import (33, 34). Accordingly, truncation of the C terminus of CPSF6 leads to increased cytoplasmic expression and inhibition of HIV-1 nuclear entry and infection (21, 33) in a TNPO3-dependent manner (35, 36). Disruption of CPSF6 binding to HIV-1 capsid is mediated by alteration of CPSF6 at amino acid F321 in the central proline-rich domain (37) or by mutations in CA, including residues 57, 70, 74, 77, and 105 (21, 32, 38, 39). While reduction of CPSF6 expression or loss of capsid binding to CPSF6 in cells does not affect overall HIV-1 infection in most cell types, viral DNA integration is mistargeted outside gene-dense, transcriptionally active host chromatin to heterochromatic lamina-associated domains (28, 40, 41).

In this study, we investigated whether HIV-1 capsid interacts with CPSF6 in the host cell cytoplasm and whether this interaction affects capsid trafficking and subsequent virus infectivity in different cell types. Using live-cell microscopy, we visualized wild-type (WT) HIV-1 complexes colocalized with cytoplasmic CPSF6 that trafficked together on microtubules. By negative-stain transmission electron microscopy (TEM), we show that purified CPSF6 protein forms oligomers that bind and disrupt CA tubular assemblies. Inhibiting HIV-1 capsid interaction with CypA led to increased association of viral particles or *in vitro* CA assemblies with CPSF6 and changes in WT HIV-1 complex trafficking that corresponded to reduced infectivity. Depletion of CPSF6 affected capsid trafficking, albeit differentially depending on the cell type.

## RESULTS

### CPSF6 is expressed in the perinuclear region and traffics on microtubules with WT HIV-1 complexes.

**FIG 1:**
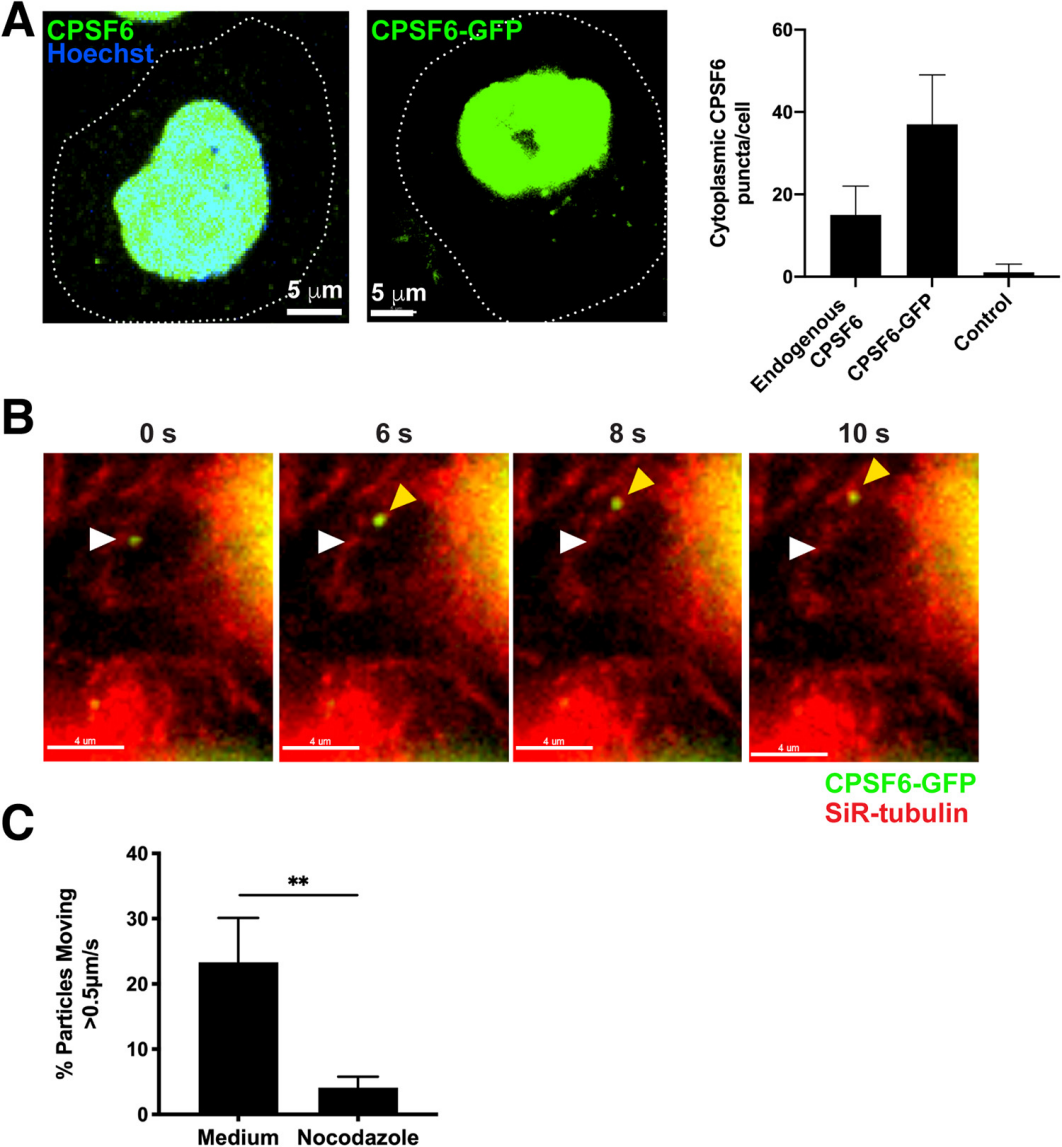
CPSF6 puncta are detected in the perinuclear region and traffic on microtubules. (A) Endogenous CPSF6 stained with antibody or expression of CPSF6-GFP is shown in HeLa cells (dotted lines, cell outlines). CPSF6 is expressed as two different isoforms composed of 551 or 588 amino acid residues; exogenously expressed proteins throughout this study were based on the 588 isoform. Quantification of endogenous cytoplasmic CPSF6 puncta by antibody staining (*n* = 103) or CPSF6-GFP expression (*n* = 96) was performed and compared to control staining (*n* = 53) in HeLa cells. (B) Movement of a CPSF6-GFP higher-order complex (green) is shown in a HeLa cell stained with SiR-tubulin (red) by HILO live-cell imaging. The white arrow indicates the location of the complex at the first time point; the yellow arrow indicates the location at subsequent time points, and the white line indicates the trajectory. (See also Movie S1.) (C) The percentage of cytoplasmic CPSF6-GFP complexes that trafficked >0.5 μm/s in HeLa cells treated with or without nocodazole. Error bars indicate standard deviations (STDEV) of *n* ≥ 185 complexes.

As CA protein may dissociate from HIV-1 nucleic acid complexes prior to entry into the nucleus where CPSF6 predominantly is expressed, we examined whether CPSF6 was expressed in the cell cytoplasm. Antibody staining of endogenous CPSF6 (NBP1-85676; Novus) or expression of green fluorescent protein (GFP)-tagged CPSF6 (CPSF6-GFP) in HeLa cells showed mostly nuclear expression as well as punctate cytoplasmic expression mainly near the nuclear membrane, which may indicate higher-order complex formation (Fig. 1A). Highly inclined and laminated optical sheet (HILO) live-cell microscopy enabled precise tracking of rapidly moving fluorescent complexes at high temporal resolution with relatively low photobleaching. Perinuclear CPSF6-GFP puncta were shown to be dynamic in cells with linear movement and were colocalized with microtubules (Fig. 1B, Movie S1). Inhibition of microtubule polymerization with nocodazole inhibited CPSF6-GFP movement in cells, suggesting that CPSF6 traffics on microtubules itself or by binding another host protein (Fig. 1C).

10.1128/mBio.03142-20.7 MOVIE S1 CPSF6-GFP traffics on microtubules. Live-cell HILO imaging is shown of a CPSF6-GFP (green) higher-order complex trafficking on a microtubule stained with SiR (red) in a HeLa cell. Download Movie S1, MOV file, 0.5 MB. Copyright © 2021 Zhong et al. 2021 Zhong et al. https://creativecommons.org/licenses/by/4.0/ This content is distributed under the terms of the Creative Commons Attribution 4.0 International license.

**FIG 2:**
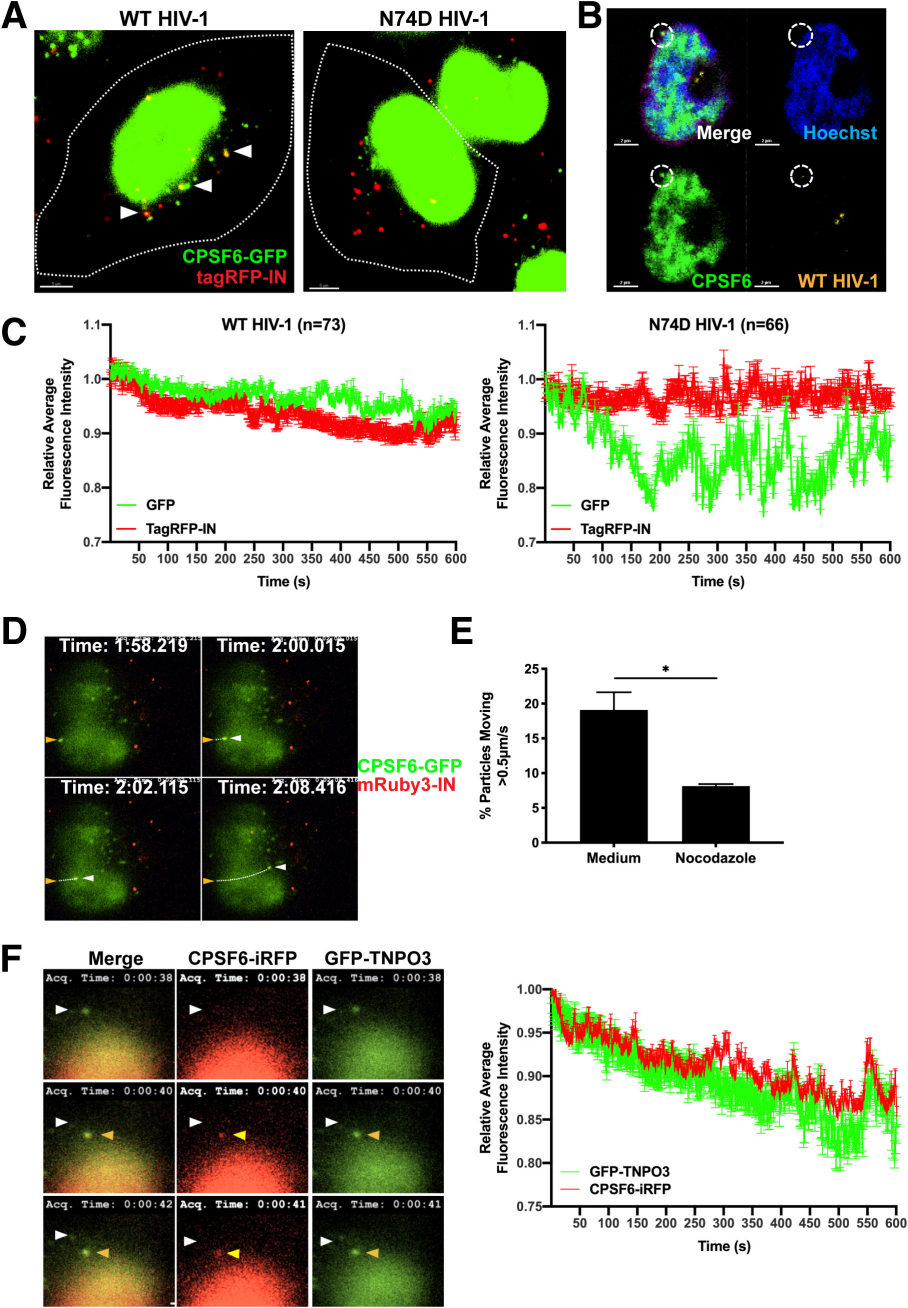
HIV-1 complexes associate with perinuclear CPSF6 and cotraffic on microtubules. (A) Confocal images of HeLa cells (7.5 × 10^5^) expressing CPSF6-GFP (green) are shown 60 min after infection with 10 ng p24 equivalent of WT HIV-1 or N74D HIV-1 containing tagRFP-IN (red). White arrows indicate tagRFP-IN particles colocalized with CPSF6-GFP (yellow). (B) Confocal micrographs show a representative SupT1 cell infected with WT HIV-1 containing mRuby3-IN (yellow) and stained with Hoechst (blue) and antibodies against endogenous CPSF6 (green). The white circle indicates cytoplasmic mRuby3-IN and CPSF6 colocalization. (C) The average intensities of cytoplasmic tagRFP-IN from WT (*n* = 73) or N74D (*n* = 66) virus complexes and CPSF6-GFP during HILO imaging were measured for 10 min, normalized to 1.0 at time zero, and graphed. Error bars indicate STDEV. (D) The track of a WT HIV-1 mRuby3-IN complex (red) colocalized with CPSF6-GFP (green) is shown by HILO imaging in a HeLa cell over time (see Movie S3). The yellow arrow indicates the location of the complex at the first time point, and white arrows indicate locations at subsequent time points. (E) The percentage of cytoplasmic CPSF6-GFP complexes colocalized with mRuby3-IN (*n* = 156 to 189) that trafficked >0.5 μm/s in HeLa cells treated with or without nocodazole. Error bars indicate standard errors of the mean (SEM). (F) A HeLa cell expressing GFP-TNPO3 (green) and CPSF6-iRFP (red) is shown. The white arrow indicates the location of a GFP-TNPO3 complex at the first time point that became colocalized with CPSF6-iRFP at subsequent time points (yellow arrow; see Movie S4). The average intensities of cytoplasmic GFP-TNPO3 and CPSF6-iRFP complexes (*n* = 96) during HILO imaging were measured for 10 min, normalized to 1.0 at time zero, and graphed. Error bars indicate the STDEV.

As HIV-1 RTCs also traffic on microtubules (42) (Movie S2), we examined the association of fluorescently labeled HIV-1 particles with cytoplasmic CPSF6-GFP in cells. Vesicular stomatitis virus glycoprotein (VSV-G)-pseudotyped WT or N74D HIV-1 encoding firefly luciferase and labeled with integrase (IN) tagged with the fluorophore mRuby3 or tagRFP was used at equal amounts (10 ng p24) to infect cells expressing CPSF6-GFP. Labeled IN colocalized with viral RNA in particles or with viral RNA and CA protein early after infection, the latter of which demarcates RTCs/PICs (43). WT complexes were colocalized with perinuclear CPSF6-GFP in HeLa cells, while N74D complexes were not (Fig. 2A). WT HIV-1 complexes were also colocalized with endogenous cytoplasmic CPSF6 in SupT1 CD4^+^ T cells (Fig. 2B). Multiple WT or N74D virus particles that colocalized with CPSF6-GFP were assessed by live-cell imaging over time. The fluorescence intensity of WT HIV-1 complexes remained associated with CPSF6-GFP, whereas the fluorescence intensity of N74D viral particles did not (Fig. 2C). This is consistent with a recent study showing WT HIV-1 particles associated with CPSF6 initially outside the nucleus (14). WT HIV-1 particles associated with CPSF6-GFP trafficked rapidly and linearly, suggestive of microtubule movement (Fig. 2D, Movie S3). Consistent with this interpretation, trafficking of WT HIV-1 particles associated with CPSF6-GFP was inhibited with nocodazole treatment (Fig. 2E). Finally, CPSF6 tagged with iRFP670 trafficked toward the nucleus with its TNPO3 binding partner, which was tagged with GFP (Fig. 2F, Movie S4). These results suggest that HIV-1 complexes traffic with CPSF6 on microtubules.

10.1128/mBio.03142-20.8 MOVIE S2 HIV-1 complexes traffic on microtubules. Live-cell HILO imaging of mRuby3-IN particles (red) with SiR-stained microtubules (white) in a HeLa cell after infection with WT HIV-1. Download Movie S2, MOV file, 1.0 MB. Copyright © 2021 Zhong et al. 2021 Zhong et al. https://creativecommons.org/licenses/by/4.0/ This content is distributed under the terms of the Creative Commons Attribution 4.0 International license.

10.1128/mBio.03142-20.9 MOVIE S3 HIV-1 complexes associate and traffic with CPSF6-GFP in the cytoplasm. Live-cell HILO imaging from the bottom of a HeLa cell expressing CPSF6-GFP (green) and infected with WT HIV-1 containing mRuby3-IN (red). Download Movie S3, MOV file, 2.5 MB. Copyright © 2021 Zhong et al. 2021 Zhong et al. https://creativecommons.org/licenses/by/4.0/ This content is distributed under the terms of the Creative Commons Attribution 4.0 International license.

10.1128/mBio.03142-20.10 MOVIE S4 CPSF6-iRFP traffics with GFP-TNPO3. Live-cell HILO imaging is shown of a HeLa cell expressing CPSF6-iRFP (red) and GFP-TNPO3 (green). The left panel shows both iRFP and GFP signal, the middle panel shows iRFP only, and the right panel shows GFP only. Download Movie S4, MOV file, 4.3 MB. Copyright © 2021 Zhong et al. 2021 Zhong et al. https://creativecommons.org/licenses/by/4.0/ This content is distributed under the terms of the Creative Commons Attribution 4.0 International license.

### Changes in the CPSF6 RS domain alter WT HIV-1 complex trafficking.

**FIG 3:**
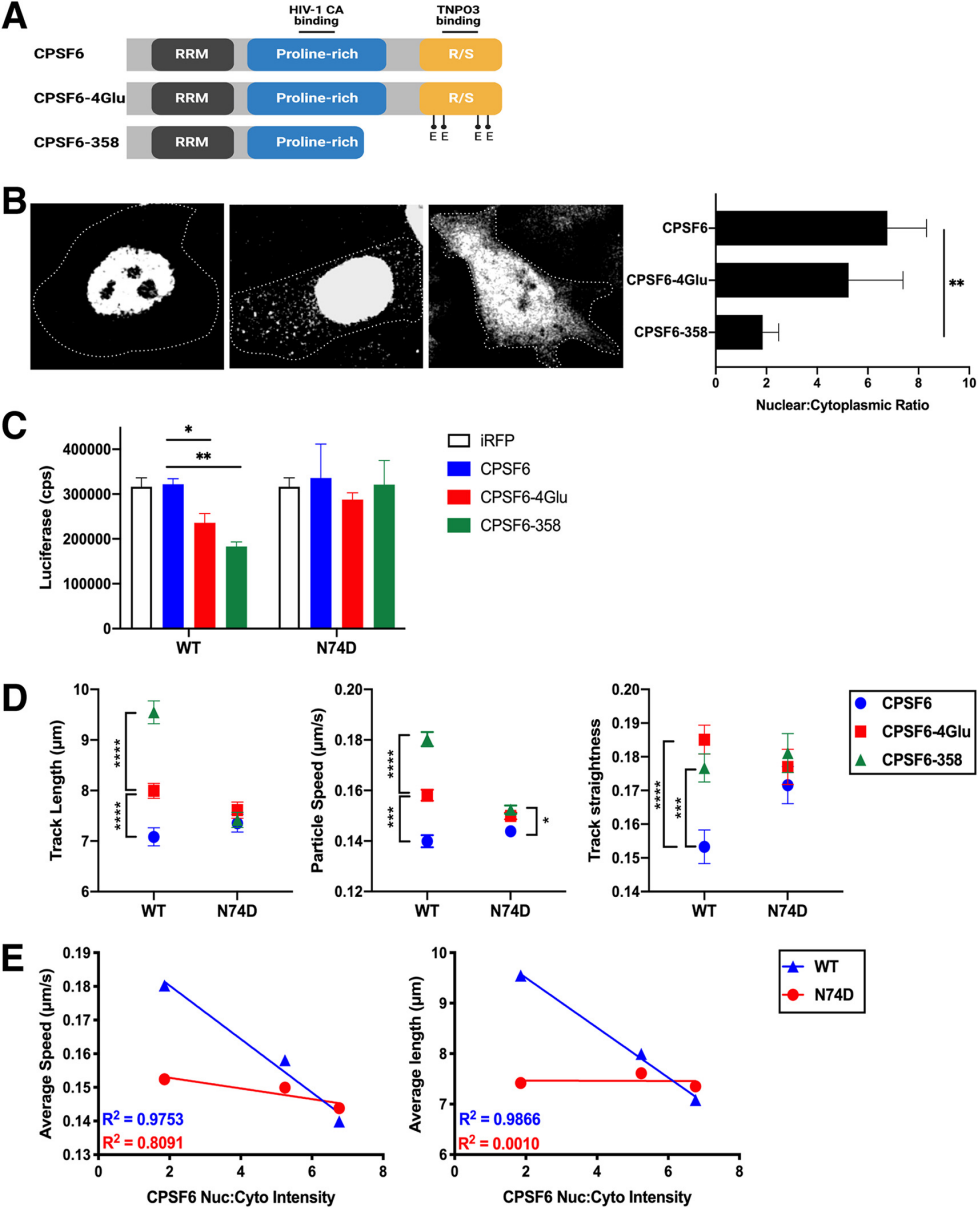
Alteration of the CPSF6 RS domain impacts HIV-1 trafficking. (A) CPSF6 protein domains and associated CPSF6-4Glu and CPSF6-358 changes. (B) Confocal microscopy images are shown of cells expressing CPSF6-iRFP, CPSF6-4Glu-iRFP, or CPSF6-358-iRFP. Cell peripheries are indicated by dotted lines. The graph shows the nuclear to cytoplasmic ratio of CPSF6 for each construct (*n* = 25). (C) Infection of WT and N74D HIV-1 in HeLa cells expressing iRFP, CPSF6-iRFP, CPSF6-4Glu-iRFP, or CPSF6-358-iRFP is shown as luciferase expression (counts per second, or cps) from 2 representative independent experiments. Error bars indicate the STDEV of duplicates. (D) The average particle speeds, track lengths, and track straightness of WT or N74D HIV-1 mRuby3-IN complexes in HeLa cells expressing CPSF6-iRFP, CPSF6-4Glu-iRFP, or CPSF6-358-iRFP are shown from one of two independent HILO live-cell imaging experiments (see also Fig. S1). Error bars indicate the SEM. (E) Correlations of the average particle speed or average track length and CPSF6 nuclear:cytoplasmic ratio are shown for WT and N74D HIV-1.

Truncation of CPSF6 at residue 358 (CPSF6-358), which removes the RS domain, or alteration of four positively charged amino acids (K547, R549, R559, and R561) in the RS domain to glutamic acid (CPSF6-4Glu) (Fig. 3A) leads to decreased nuclear localization of CPSF6 and restriction of WT HIV-1 infection at the step of nuclear entry (21, 33). Expression of iRFP670-tagged CPSF6-4Glu or CPSF6-358 led to increased cytoplasmic localization in cells, with the truncated protein showing a greater effect than the 4Glu mutant, which revealed greater variability in cytoplasmic expression levels than the other constructs (Fig. 3B). Similarly, expression of CPSF6-358 resulted in greater restriction of WT HIV-1 infection than expression of CPSF6-4Glu (Fig. 3C). N74D HIV-1, which does not bind to CPSF6, was not restricted by either mutant.

10.1128/mBio.03142-20.1 FIG S1 Mutation or truncation of the CPSF6 R/S domain alters HIV-1 trafficking. Results from HILO live-cell imaging are shown that were summarized in Fig. 3D. (A to C) The particle speed (A), track length (B), and track straightness (C) of individual WT or N74D HIV-1 mRuby3-IN complexes in HeLa cells expressing CPSF6-iRFP, CPSF6-4Glu-iRFP, or CPSF6-358-iRFP are shown. Error bars indicate the SEM. Dotted lines denote the mean of WT complexes in CPSF6-iRFP cells. The numbers (*n*) of mRuby3-IN complexes analyzed for each condition are listed at the bottom. Download FIG S1, JPG file, 0.2 MB. Copyright © 2021 Zhong et al. 2021 Zhong et al. https://creativecommons.org/licenses/by/4.0/ This content is distributed under the terms of the Creative Commons Attribution 4.0 International license.

To determine if relocalization of CPSF6 to the cytoplasm affects HIV-1 complex trafficking, live-cell imaging of WT and N74D HIV-1 complexes was performed in cells expressing fluorescently tagged CPSF6, CPSF6-4Glu, or CPSF6-358. To avoid imaging particles that have not yet fused out of endosomes into the cytoplasm, imaging was performed using mRuby3-IN-labeled WT HIV-1 particles that were also labeled with the glycosylphosphatidylinositol targeting motif of decay-accelerating factor (44) tagged with fluorogen-activating protein (FAP-GPI) to label the virus membrane. Loss of FAP-GPI signal from the mRuby3 signal signified that the HIV-1 membrane had fused with the endosome and the contents of the virus were released into the cytoplasm. During synchronized infection, we observed that nearly all mRuby3 signal separated from the viral membrane by 50 min (data not shown). Thus, for virus tracking experiments, acquisition of images began at 60 min postinfection.

Fluorescent IN complexes were analyzed for average speed, track length (distance), and track straightness (calculated by the distance between the end points over the trajectory length; also known as displacement). In HeLa cells expressing CPSF6-GFP, WT and N74D viral complexes had similar speeds and track lengths but differed in track straightness (Fig. 3D, Fig. S1), suggesting that both capsids utilize microtubules but differences in binding host proteins affect microtubule movement. WT HIV-1 particle movement in cells expressing CPSF6-GFP did not differ from normal HeLa cells (data not shown). In contrast, WT HIV-1 particles increased in all three measurements when CPSF6-4Glu-iRFP670 or CPSF6-358-iRFP670 was expressed in cells (Fig. 3D, Fig. S1). Little or no change in particle speed, track length, or track straightness was observed for N74D complexes in the presence of CPSF6-4Glu or CPSF6-358. Similar to the effect of increased CPSF6 cytoplasmic localization on WT HIV-1 infectivity, average WT virus particle speed and track length inversely correlated with the intensity of nuclear CPSF6 expression (Fig. 3E). These data suggest that HIV-1 complex trafficking is altered in the cytoplasm by enhanced CPSF6 cytoplasmic localization. Furthermore, increased HIV-1 mobility is associated with an infectivity defect.

### CPSF6 oligomerizes and disrupts assembled WT CA.

Previously, we showed that purified recombinant CPSF6-358 protein formed oligomers and disrupted WT HIV-1 CA tubular assemblies *in vitro* (43). To determine whether full-length CPSF6 had similar properties, it was purified and characterized with WT and N74D CA tubular assemblies. To obtain soluble CPSF6, an N-terminal maltose binding protein (MBP) fusion construct was expressed and purified, resulting in two peaks in size exclusion chromatography (Fig. S2A, labeled P1 and P2) that corresponded to the tagged full-length CPSF6, as confirmed by Western blot analysis (Fig. S2B). This suggests that the purified fusion protein may adopt different oligomeric states similar to what was observed for CPSF6-358, which also displayed two peaks in a size exclusion chromatography profile with dimer and large oligomers (43). Removal of the MBP-tag with HRV-3C protease resulted in precipitation of CPSF6 from both P1 and P2 (Fig. S2C). Therefore, MBP-tagged soluble MBP-His_6_-CPSF6-588 (denoted here as “MBP-CPSF6”) was used for further binding experiments.

10.1128/mBio.03142-20.2 FIG S2 Purification of MBP-CPSF6 with an MBP tag from a mammalian cell expression system. (A) Gel filtration profile of the protein eluted from the Superdex 200 16/60 column. The two MBP-CPSF6 peaks are labeled P1 and P2. (B) SDS-PAGE and Western blot analysis of MBP-CPSF6 purification. Samples were taken from cell lysate, supernatant (sup), pellet, flowthrough (FT), wash, and elute 1, 2, and 3 from amylose resin, and peaks (P1 and P2) from the Superdex 200 16/60 column (shown in panel A) were stained with Coomassie blue (top) or processed with anti-MBP (middle) or anti-CPSF6 (bottom) antibody following Western blotting. (C) MBP tag removal analysis; the uncleaved P1 and P2 are shown in lanes 2 and 5, and the supernatant (s) and pellet (p) of P1 and P2 after cleavage with HRV-3C protease are shown in lanes 3, 4, 6, and 7, respectively. Samples were stained with Coomassie blue. Proteins are indicated by arrows on the right. Download FIG S2, JPG file, 0.2 MB. Copyright © 2021 Zhong et al. 2021 Zhong et al. https://creativecommons.org/licenses/by/4.0/ This content is distributed under the terms of the Creative Commons Attribution 4.0 International license.

**FIG 4:**
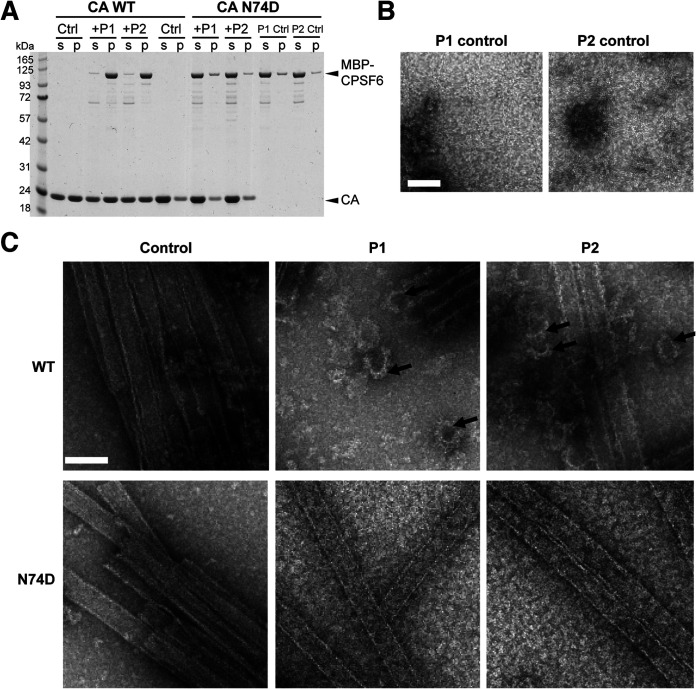
CPSF6 binds to and disrupts CA tubular assemblies. (A) SDS-PAGE analysis of WT and N74D CA assemblies following incubation with MBP-CPSF6 P1 or P2 and centrifugation. The gel was stained with Coomassie blue; supernatant (s) and pellet (p) samples are indicated. (B) Representative negative-stain EM micrographs are shown of P1 or P2 incubated in buffer. (C) Representative negative-stain EM micrographs are shown of WT CA (top panels; see also Fig. S3) and CA N74D (bottom panels) tubular assemblies alone (control) or with 9 μM MBP-CPSF6 P1 or P2. The arrows indicate capsid fragments. Scale bar, 100 nm.

Incubation of *in vitro* preassembled WT HIV-1 CA tubes with MBP-CPSF6 (both P1 and P2) resulted in cosedimentation of MBP-CPSF6/CA complexes in the pelleted fractions (Fig. 4A). The ratio of CPSF6 to WT CA in the pelleted fraction was 0.097 ± 0.005. Negligible binding of MBP-CPSF6 to N74D HIV-1 CA tubes was observed under the same assay conditions, giving a CPSF6/CA ratio of 0.006 ± 0.008. TEM of the negative stained samples showed a drastic structural disruption of capsid tubes when incubated with MBP-CPSF6 (P1 or P2), while N74D CA tubes remained intact (Fig. 4C). MBP-CPSF6, by itself, formed protein oligomers for both P1 and P2 fractions in CA assembly buffer (Fig. 4B). Binding of MBP-CPSF6 to WT CA tubes resulted in dissolution of tubes and an appearance of distinct curved capsid remnants associated with MBP-CPSF6 densities. Intriguingly, the amount of pelletable capsid did not change upon capsid disruption (Fig. 4A), suggesting that the predominant effect of MBP-CPSF6 is HIV-1 capsid fragmentation without dissociation into soluble proteins. Dose-dependent binding of MBP-CPSF6 to CA tubes was observed for both MBP-CPSF6 P1 and P2 by TEM (Fig. S3). This represents the first direct evidence of full-length CPSF6 binding to and disruption of WT CA tube assemblies.

10.1128/mBio.03142-20.3 FIG S3 Dose-dependent effect of MBP-CPSF6 on HIV-1 capsid tubes. (A to J) Representative negative-stain EM micrographs of CA with different concentrations of MBP-CPSF6 P1 or P2. WT CA tubular assemblies alone (A) or with 2 μM (B), 5 μM (C), 10 μM (D), 15 μM (E), or 18 μM (F) of MBP-CPSF6 P1 or with 2 μM (G), 5 μM (H), 10 μM (I), or 12.8 μM (J) of MBP-CPSF6 P2. Scale bars, 100 nm. (K and L) Dose-dependent effect of MBP-CPSF6 on CA tubes (K) or CA-NC tubes (L). Shown is the binding of P1 (circles) and P2 (squares) to assembled WT CA tubes or CA-NC tubes. The error bars indicate the standard deviation of the values from biological replicates. Download FIG S3, JPG file, 0.5 MB. Copyright © 2021 Zhong et al. 2021 Zhong et al. https://creativecommons.org/licenses/by/4.0/ This content is distributed under the terms of the Creative Commons Attribution 4.0 International license.

### HIV-1 induces cytoplasmic higher-order CPSF6 complex formation in a CypA-dependent manner.

**FIG 5:**
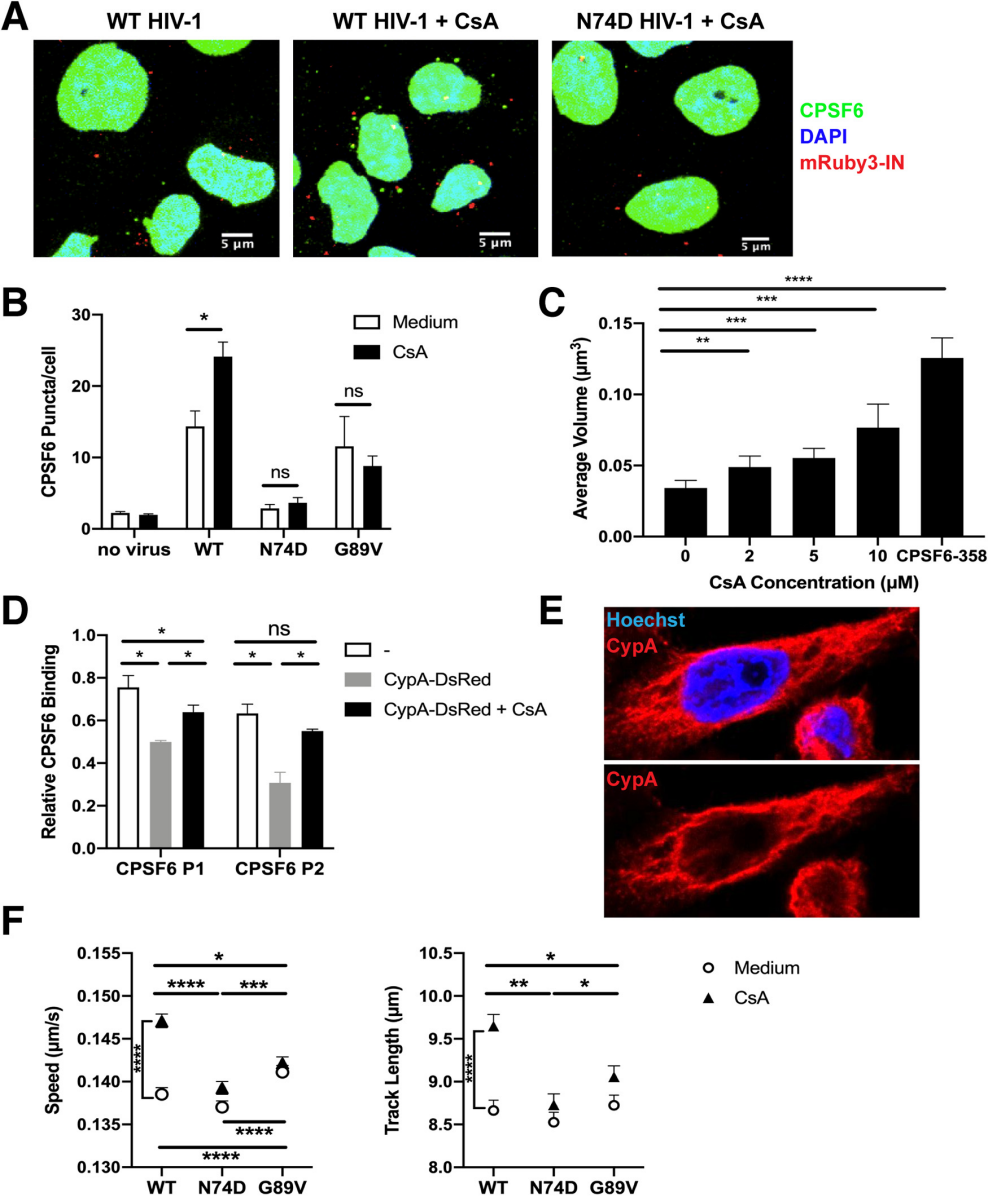
HIV-1 induction of cytoplasmic CPSF6 higher-order complex formation as a function of CypA binding. (A) Representative confocal micrographs are shown of HeLa cells stained with CPSF6 antibodies (green) and Hoechst (blue) after infection with WT HIV-1 and WT or N74D HIV-1 in the presence of 10 μm CsA. Viruses contain mRuby3-IN (red). (B) Cytoplasmic CPSF6 staining was quantified in cells (*n* ≥ 1,235) treated with or without 10 μm CsA and infected for 1 h with WT, N74D, or G89V HIV-1. (C) The average volume of cytoplasmic CPSF6 puncta (*n* ≥ 1,235) was quantified in HeLa cells treated with or without CsA. Cells expressing CPSF6-358-GFP were used as a positive control. (D) CA-NC assemblies were incubated for 1 h in buffer, with CypA-DsRed, or CypA-DsRed and CsA, and then incubated with MBP-CPSF6 P1 or P2 for 1 h. The relative binding of CPSF6 binding to CA-NC assemblies was quantified in each sample from stained SDS-PAGE gels. Error bars indicate the STDEV from 3 independent experiments. (E) A representative confocal micrograph of HeLa cells stained with Hoechst (blue) and CypA antibodies (red). (See also Fig. S5A to C.) (F) The average particle speeds and track lengths of WT, N74D, or G89V HIV-1 mRuby3-IN complexes in HeLa cells treated with or without 10 μm CsA are shown for 1 of 3 independent experiments with 719 to 5,009 particles per condition. Error bars indicate the SEM.

In cells, CPSF6-358 forms puncta around WT HIV-1 mRuby3-IN complexes early after virus entry, leading to premature capsid permeabilization (43). These did not form in the presence of a small-molecule inhibitor, PF74, which blocks CPSF6 binding to capsid (45), or if N74D HIV-1 was used. To determine if full-length higher-order CPSF6 complexes form in the cytoplasm, immunostaining of CPSF6 was performed before and after HIV-1 infection. Higher-order CPSF6 complexes were visualized in the perinuclear region after WT HIV-1 infection but not after N74D HIV-1 infection (Fig. 5A and B) and were associated with IN-containing complexes (Fig. 2) and CA (p24) staining (Fig. S4A). CPSF6 puncta were also observed in the nuclei of cells at later time points after WT HIV-1 infection (data not shown). Consistent with our *in vitro* results (Fig. 4), these data suggest that CPSF6 binds to WT capsid in the cytoplasm of infected cells.

10.1128/mBio.03142-20.4 FIG S4 CypA prevents HIV-1 capsid binding to CPSF6. (A and B) HeLa cells were infected with WT HIV-1 containing mRuby-IN or CypA-DsRed in the presence or absence of 10 μM CsA for 1 h. Cells were fixed and stained with p24 antibodies. The (A) percentage red viral complexes that were also positive for GFP and (B) total number of p24+ particles in cells are plotted. Error bars represent the STDEV. (C) SDS-PAGE of WT CA-NC assemblies were incubated with CypA-DsRed with or without CsA for 1 h at room temperature following incubation with MBP-CPSF6 P1 or P2 and then centrifuged. The gel was Coomassie blue stained, with supernatant (s) and pellet (p) sample indicated. (D) The binding ratio of CypA-DsRed with WT CA-NC assemblies. The error bars indicate the standard deviation of the values of three independent experiments. Download FIG S4, JPG file, 0.3 MB. Copyright © 2021 Zhong et al. 2021 Zhong et al. https://creativecommons.org/licenses/by/4.0/ This content is distributed under the terms of the Creative Commons Attribution 4.0 International license.

10.1128/mBio.03142-20.5 FIG S5 CypA expression is excluded from the microtubule-organizing center of HeLa cells and SupT1 cells. (A and B) Confocal micrographs are shown of (A) HeLa cells stained with Hoechst, CypA antibodies, and CPSF6 antibodies or (B) stained with Hoechst, CypA antibodies, and tubulin antibodies. (C) Confocal micrographs are shown of SupT1 cells stained with Hoechst, CypA antibodies, and tubulin antibodies. Arrows show perinuclear exclusion of CypA. Circles show the microtubule organizing center (MTOC). (D) HeLa cells were treated with 5 mM CsA and infected with WT, N74D, or G89V HIV-1. Results are shown as the mean luciferase expression of duplicates, with error bars indicating the STDEV. Download FIG S5, JPG file, 0.3 MB. Copyright © 2021 Zhong et al. 2021 Zhong et al. https://creativecommons.org/licenses/by/4.0/ This content is distributed under the terms of the Creative Commons Attribution 4.0 International license.

Our previous work demonstrated that higher-order CPSF6-358 complexes were larger and formed more rapidly when CypA binding to CA was inhibited by CsA (43). Therefore, we examined whether the same would be true for full-length CPSF6. Indeed, when cells were treated with CsA and infected with WT HIV-1, greater numbers of CPSF6 higher-order complexes were observed (Fig. 5A and B). The volume of the complexes that formed in the presence of WT virus increased with increasing concentrations of CsA (Fig. 5C), suggesting that inhibiting more CypA binding allowed more CPSF6 to bind to HIV-1 capsid. In contrast, CPSF6 complex formation was indistinguishable from background after infection with N74D HIV-1 (Fig. 5A and B). To confirm that the loss of CypA binding to capsid was responsible for the increase in CPSF6 higher-order complexes, cells were infected with HIV-1 CA mutant G89V, which is defective for CypA binding (23). Because G89V HIV-1 is restricted by CPSF6-358 and thus can still bind CPSF6 (35), we expected that this virus would induce CPSF6-GFP puncta that would not increase in number in the presence of CsA, which is what was observed (Fig. 5B).

As removal of CypA from HIV-1 capsid enhanced CPSF6 higher-order complex formation, we hypothesized that CPSF6 complex formation may be prevented if CypA binding to capsid was enhanced. Thus, virus was produced in the presence of CypA-DsRed, an oligomeric form of fluorescently labeled CypA with higher avidity to HIV-1 capsid than unlabeled CypA (46). Cells were infected with WT HIV-1 labeled with CypA-DsRed or with mRuby3-IN in the presence or absence of CsA and stained for CA (p24; Fig. S4B). Similar levels of p24 staining were observed under all conditions. Virus containing mRuby3-IN led to formation of many CPSF6-358-GFP puncta associated with p24 that increased with CsA treatment (Fig. S4A). However, cells infected with CypA-DsRed-labeled HIV-1 had significantly fewer GFP puncta, and CsA treatment did not increase their formation, suggesting that enhanced CypA binding to capsid prevents HIV-1 interaction with CPSF6-358 and, likely, also CPSF6.

To directly test the ability of CypA to shield CPSF6 binding to HIV-1 capsid, MBP-CPSF6 protein (P1 and P2) binding to nanotubes composed of recombinant WT CA-SP1-nucleocapsid (CA-NC) protein was quantified in the presence or absence of CypA-DsRed and in the presence or absence of CsA. (Fig. S4C). Binding of CypA-DsRed to CA-NC tubes was not affected by subsequent MBP-CPSF6 binding but was inhibited by CsA treatment (Fig. S4D). MBP-CPSF6 binding to HIV-1 CA-NC tubes decreased when CypA-DsRed was already bound, effects that were rescued partially (P1) or completely (P2) by the presence of CsA (Fig. 5D). Collectively, these data demonstrate that CypA binding to capsid prevents CPSF6 from binding.

### Loss of CypA binding leads to altered cytoplasmic trafficking of WT HIV-1 complexes in a CPSF6-dependent manner.

Although CypA is packaged into virions, HIV-1 capsid interactions with target cell CypA modulate infectivity (24). In contrast to CPSF6 expression, endogenous CypA localized to the cytoplasm of HeLa cells with somewhat of a filamentous appearance (Fig. 5E, Fig. S5A). Interestingly, not only was CypA excluded from the nucleus, but its expression was absent from portions of the perinuclear region (Fig. S5A) that corresponded to the microtubule-organizing center (Fig. S5B). Similar expression of CPSF6 and CypA was observed in SupT1 CD4^+^ T cells (Fig. S5C).

To determine whether the loss of CypA binding to HIV-1 capsid could affect virus trafficking, live-cell microscopy was performed on WT, N74D, and G89V HIV-1 in the presence and absence of CsA in HeLa cells (Fig. 5F) or HeLa cells expressing CPSF6-GFP (data not shown). In the absence of drug, the average speed and track length of viral complexes were similar for WT HIV-1 and N74D HIV-1, while G89V HIV-1 complexes trafficked significantly faster and had similar track lengths. As observed for WT HIV-1 trafficking in cells expressing mutant CPSF6 (Fig. 5), the higher rate of speed of G89V viral complexes was associated with lower infectivity (Fig. S5D). However, CsA treatment led to significantly increased speed and track length of WT HIV-1 particles. CsA did not affect N74D or G89V viral particles. These results suggest that the loss of CypA binding to WT capsid influences trafficking of HIV-1 complexes in the cytoplasm in a CPSF6-dependent manner, as N74D viral particles were not affected by CsA treatment. G89V complexes that bind to CPSF6 but not CypA had altered trafficking and lower infectivity irrespective of CsA treatment. The expression and trafficking data together suggest that CypA prevents virus cores from binding prematurely to CPSF6 during trafficking to the nucleus.

### Depletion of CPSF6 rescues the HIV-1 complex trafficking defect caused by loss of CypA binding.

**FIG 6:**
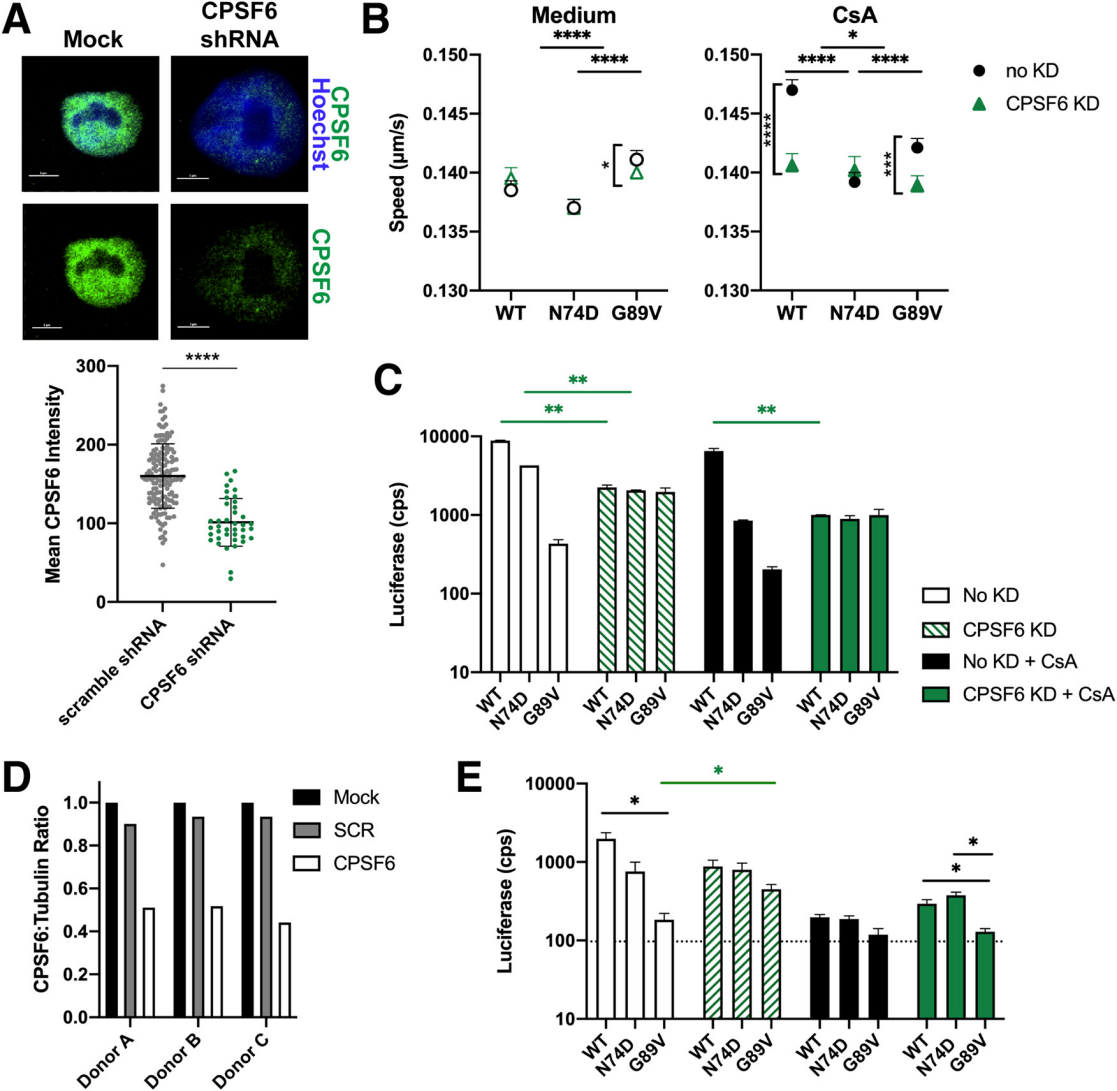
CPSF6 knockdown in HeLa cells and primary PBMC alters HIV-1 trafficking and decreases infection in the absence of CypA binding. (A) Representative confocal micrographs are shown for CPSF6 antibody (green) and Hoechst (blue) staining in HeLa cells with and without transduction with lentivirus expressing CPSF6 shRNA. The white bars indicate 5 μm. The mean intensity of CPSF6 antibody staining is shown from individual HeLa cells transduced with lentiviruses expressing scrambled (SCR) or CPSF6 shRNA. The error bars indicate the STDEV (*n* ≥ 157 cells). (B) The average particle speeds of WT, N74D, or G89V HIV-1 mRuby3-IN complexes in HeLa cells expressing SCR or CPSF6 shRNA and treated with or without 10 μm CsA are shown, representative of 2 independent experiments with 2,239 to 5,009 particles per condition. Error bars indicate the SEM. (C) The infection of WT, N74D, or G89V HIV-1 in the same cells as shown in panel B are graphed. Error bars indicate the STDEV of duplicates. (D) CPSF6 depletion was quantified by the ratio of CPSF6 to α-tubulin measured by Western blot analysis for activated PBMCs from each donor. (See also Fig. S6A.) (E) Representative infections with WT, N74D, and G89V HIV-1 were measured in primary PBMC cells from a donor after shRNA depletion of CPSF6, treatment with CsA, or both. (See also Fig. S6B.)

To validate that the effect of CsA treatment on WT viral complex trafficking is mediated by CPSF6, CPSF6 was depleted from cells using short hairpin RNA (shRNA) knockdown. HeLa cells were transduced with lentiviruses expressing a shRNA targeting CPSF6 or a scrambled control shRNA. Knockdown (KD) of CPSF6 was verified by immunofluorescence staining (Fig. 6A). In the absence of CsA, CPSF6 KD had no effect on WT virus particle tracking (Fig. 6B). Infectivity of WT HIV-1 in untreated cells decreased after CPSF6 depletion (Fig. 6C), which may be attributable to previously described reduced cell proliferation of CPSF6 knockout cells (40). As shown above (Fig. 5F), CsA led to a significant increase in WT HIV-1 complex speed and track length in HeLa cells (Fig. 6B).

10.1128/mBio.03142-20.6 FIG S6 CPSF6 knockdown in primary PBMC. (A) CPSF6 depletion by shRNA was visualized in stimulated PBMCs by Western blotting of stimulated PBMCs. A scrambled shRNA was used as a knockdown control, and α-tubulin was used as a loading control for the gel. Lane 1, no shRNA; lane 2, scrambled shRNA; lane 3, CPSF6 shRNA. (B) Infections with WT, N74D, and G89V HIV-1 were measured in primary PBMC cells from 2 donors after shRNA depletion of CPSF6, treatment with CsA, or both. Download FIG S6, JPG file, 0.2 MB. Copyright © 2021 Zhong et al. 2021 Zhong et al. https://creativecommons.org/licenses/by/4.0/ This content is distributed under the terms of the Creative Commons Attribution 4.0 International license.

The CA mutants showed different cytoplasmic trafficking patterns compared to WT HIV-1. N74D complex trafficking was unaffected by CPSF6 KD and/or CsA treatment (Fig. 6B). Interestingly, depletion of CPSF6 led to a significant decrease in G89V HIV-1 complex trafficking with or without CsA treatment (Fig. 6B), which corresponded to a rescue of the infectivity defect of this mutant (Fig. 6C). Our data indicate that CypA alters HIV-1 trafficking in a CPSF6-dependent manner, further suggesting that CypA binding protects HIV-1 capsid from binding too prematurely to or too much of CPSF6.

As the HeLa cell model may not fully recapitulate HIV-1 trafficking or infection in primary CD4^+^ T cells, infectivity of WT HIV-1 and CA mutants was performed in phytohemagglutinin (PHA)-stimulated primary human peripheral blood mononuclear cells (PBMC) from 3 donors. Cells were infected in the presence or absence of CPSF6 depletion and CsA treatment. Depletion of CPSF6 was verified by Western blot quantification (Fig. 6D, Fig. S6A). Similar to the HeLa cell data, infection of N74D and G89V HIV-1 was inhibited in primary PBMC (Fig. 6E, Fig. S6B), as previously reported (21, 47). Depletion of CPSF6 partially rescued G89V HIV-1 infectivity (Fig. 6E, Fig. S6B). Although the luciferase values were comparatively low for this mutant, the data reproducibly trended with what was observed in HeLa cells (Fig. 6C).

### Depletion of CPSF6 in macrophages results in decreased HIV-1 infectivity independent of CypA binding.

**FIG 7:**
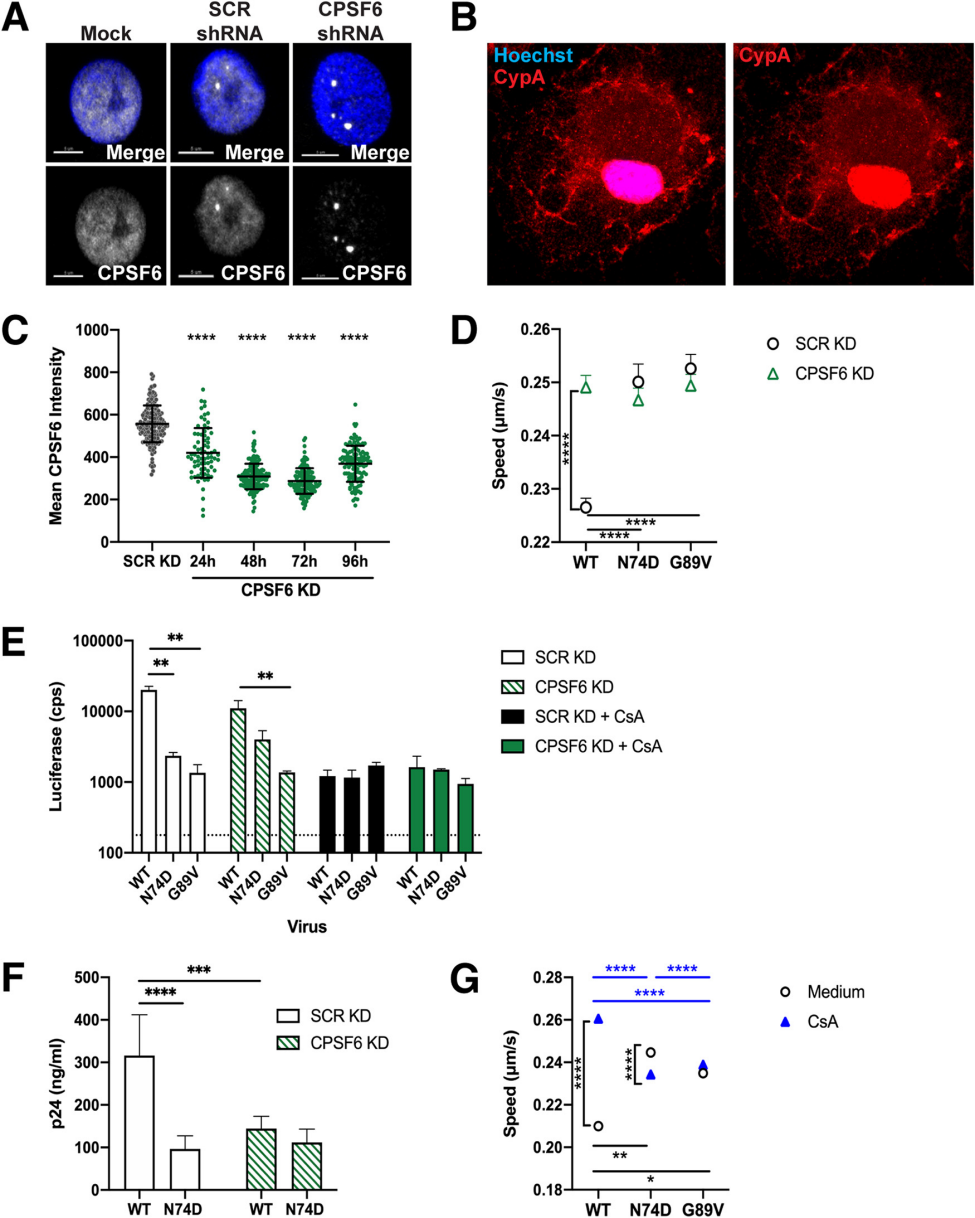
CPSF6 KD decreases HIV-1 infection in MDM. (A) Representative confocal micrographs are shown for CPSF6 antibody (white) and Hoechst (blue) staining in MDM without transduction or after transduction with lentiviruses expressing SCR shRNA or CPSF6 shRNA. The white bars indicate 5 μm. (B) A representative confocal micrograph of an MDM stained with Hoechst (blue) and CypA (red) is shown. (C) The mean intensity of CPSF6 antibody staining is shown from individual MDM transduced with lentiviruses expressing SCR or CPSF6 shRNA. The error bars indicate the STDEV of *n* ≥ 77 cells. (D) The average particle speeds of WT, N74D, or G89V HIV-1 mRuby3-IN complexes in MDM from 1 of 3 individual donors treated with scrambled shRNA (SCR KD) or CPSF6 shRNA are shown. Error bars indicate the SEM. (E) The infection of WT, N74D, or G89V HIV-1 in the same cells as shown in panel B are graphed. Error bars indicate the STDEV of duplicates, and the dotted line represents the average luciferase expression of uninfected cells. (F) WT and N74D HIV-1_NL4-BAL_ virus production of duplicate MDM infections was measured with p24 ELISA on day 8 postinfection. Results are shown as means ± the STDEV. (G) The average particle speeds of WT, N74D, or G89V HIV-1 mRuby3-IN complexes in MDM from a representative donor treated with or without CsA are shown. Error bars indicate SEM.

As we and others previously showed that N74D HIV-1 has an early infectivity defect in monocyte-derived macrophages (MDM) (28, 48), we investigated whether intracellular trafficking of HIV-1 is dependent on CPSF6 and CypA. MDM were stained for endogenous CypA and CPSF6 expression. As in HeLa cells, CPSF6 expression was predominantly nuclear in MDM (Fig. 7A). However, infection with WT HIV-1 failed to induce higher-order CPSF6 complex formation in the cytoplasm (data not shown), consistent with previous results (49) and indicative of less cytoplasmic CPSF6 expression in MDM than in HeLa cells. In contrast, CypA expression differed greatly between cell types. MDM had pronounced nuclear CypA expression in addition to patches of plasma membrane and cytoplasmic expression (Fig. 7B).

CPSF6 was depleted with shRNA in MDM, which was confirmed by immunostaining (Fig. 7A and C). Trafficking of mRuby3-IN complexes was evaluated in MDM from 3 donors with and without CPSF6 depletion. CPSF6 depletion led to a significant increase in speed and track length of WT HIV-1 complexes (Fig. 7D). Loss of CPSF6 led to a modest decrease in WT HIV-1 single-cycle infectivity, with only donor 2 being significant (Fig. 7E). However, CPSF6 depletion led to a significant decrease in spreading infection with WT HIV-1 but not with N74D HIV-1 in MDM (Fig. 7F). In normal MDM, both N74D and G89V HIV-1 had significantly decreased infectivity, which corresponded to faster mRuby3-IN trafficking (Fig. 7E). CPSF6 depletion had no effect on the trafficking of mutant CA complexes or subsequent infection. These results suggest that loss of CPSF6 in macrophages affects WT HIV-1 CA trafficking, leading to decreased infectivity.

CsA treatment of MDM significantly increased WT HIV-1 complex trafficking speed (Fig. 7G), with a corresponding decrease in infection in all donors (Fig. 7E). CPSF6 KD with CsA treatment (i.e., loss of CPSF6 and CypA binding) led to a significant increase in infectivity of WT virus in all donors compared to CsA treatment alone. N74D HIV-1 infection was significantly reduced during CsA treatment, with or without CPSF6 KD. Although the loss of CypA binding significantly reduces virus infectivity, these results suggest that alteration of HIV-1 trafficking and infectivity by depletion of CPSF6 is independent of CypA binding in macrophages.

## DISCUSSION

HIV-1 capsid has been shown to interact with many host cell factors (17). Included in this list is CPSF6, which is involved in mRNA cleavage and polyadenylation in the nucleus (21). In this study, we detected cytoplasmic, punctate expression of endogenous or fluorescently tagged CPSF6 in the perinuclear region of cells. Full-length MBP-CPSF6 protein formed oligomers *in vitro* that bind to HIV-1 CA assemblies, similar to what we previously reported for CPSF6-358 (43), which lacks the RS domain but retains the central proline-rich domain that mediates CA binding. In cells, CPSF6 puncta likely represent higher-order complexes that bind to HIV-1 capsid in the cytoplasm after viral entry. This is consistent with a recent study in which examples of CPSF6 associated with HIV-1 complexes in the cytoplasm were shown prior to nuclear import (14). Removal or truncation of the CPSF6 RS domain leads to mislocalization of CPSF6 to the periphery of the cell due to loss of TNPO3 binding and reduced HIV-1 nuclear import and infectivity (21, 33, 35). Here, we demonstrate with live-cell imaging that HIV-1 complexes trafficked with CPSF6 in a capsid-dependent manner. In addition, virus complexes increased in speed in the presence of CPSF6 RS mutants or by introducing the CA N74D mutation that abolishes CPSF6 binding. Interestingly, increased microtubule trafficking of HIV-1 was associated with reduced infectivity. Previously, we showed significant colocalization of CPSF6-358 to mRuby3-IN complexes in the cytoplasm, likely due to a high concentration at the cell periphery that was not seen with full-length CPSF6 (43). It is possible that overall faster and longer trafficking is due to CPSF6-mediated modulation of capsid integrity, which may alter the accessibility of capsid to other host proteins, such as certain microtubule motor proteins or motor adaptors that can alter cargo trafficking speed, track length, and bidirectional transport (50).

Previously, it was shown that HIV-1 capsid traffics on microtubules on its way to the nucleus (42) and that CA binds key NPC component proteins (17). HIV-1 capsid uncoating is delayed by destabilization of microtubules or knockdown of the microtubule motor proteins kinesin and dynein (5, 6), suggesting that microtubule trafficking and NPC binding are linked to capsid uncoating. Here, we demonstrate that HIV-1 capsid trafficked with CPSF6 and TNPO3 on microtubules and that CPSF6 facilitated CA tubular disassembly. Our previous work demonstrated that CPSF6-358 associates with HIV-1 complexes in a CA-dependent manner and leads to more rapid uncoating kinetics and reduced virus infectivity (43). Therefore, CPSF6 may play a role in both HIV-1 capsid uncoating, which may initiate in the cytoplasm, and nuclear import (49, 51).

**FIG 8:**
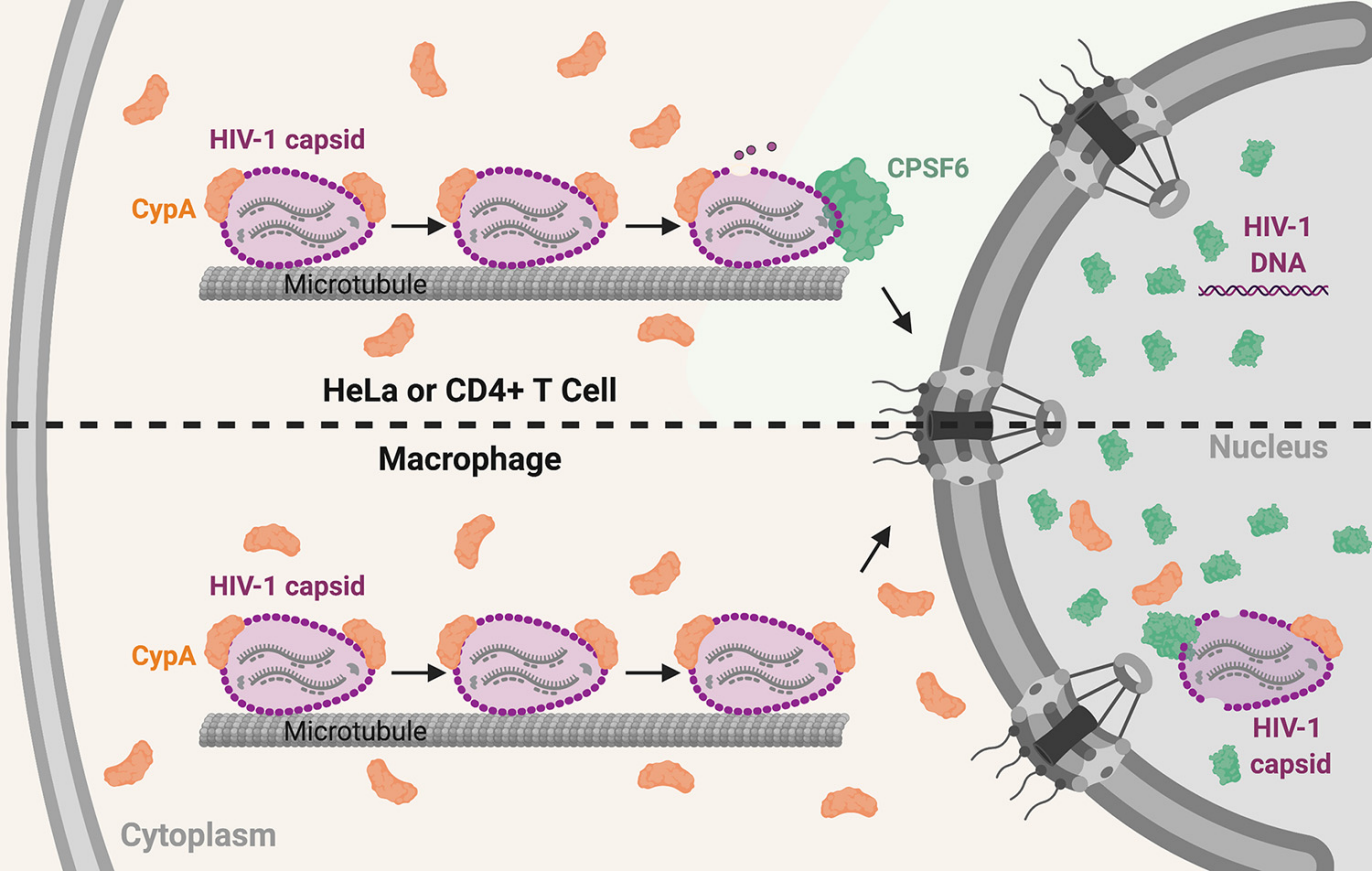
Model of CypA and CPSF6 interaction with HIV-1 capsid in HeLa/CD4^+^ T cells and macrophages. The upper condition represents the situation in HeLa and CD4^+^ T cells, where CypA regulates premature engagement of the HIV-1 capsid by cytoplasmic CPSF6. In MDM (lower scheme), HIV-1 infection does not induce higher-order CPSF6 formation in the cytoplasm. Nuclear CypA could in theory play a greater role in regulating capsid interactions and uncoating in this cell type.

CypA binding to HIV-1 capsid was described nearly 3 decades ago (18), yet until recently, its role in HIV-1 infection was ill defined. Here, we show that the loss of CypA binding to HIV-1 capsid in infected cells due to CsA treatment coincided with increased capsid binding to CPSF6, which is consistent with our previous results with fluorescently labeled CPSF6-358 (43). Conversely, production of HIV-1 in the presence of CypA-DsRed, which has increased binding to capsid compared to untagged CypA (46), reduced CPSF6 binding to capsid. This was also seen *in vitro* in a competitive binding assay with CA-NC assemblies in the presence of CypA-DsRed and MBP-CPSF6 proteins. Trafficking of HIV-1 complexes in cells increased in the presence of CsA or with the G89V CA mutation, which correlated with decreased infectivity. These results suggest that CypA binding to capsid prevents CPSF6 binding. As CypA expression in HeLa cells was restricted to the cell periphery, from which CPSF6 was excluded, we hypothesize that CypA interacts with HIV-1 capsid in the cell periphery first to prevent CPSF6 binding to HIV-1 capsid before the perinuclear region, ensuring that uncoating does not occur prematurely (Fig. 8).

Depletion of CPSF6 in cells did not affect HIV-1 infection in HeLa cells or PBMC, as previously demonstrated (21), nor did it alter HIV-1 trafficking to the nucleus in HeLa cells. As N74D HIV-1 was associated with microtubules and trafficked in a linear fashion like WT HIV-1, we suggest that HIV-1 microtubule trafficking and nuclear import can be independent of CPSF6. Surprisingly, the loss of CPSF6 expression affected WT and G89V HIV-1 trafficking during CsA treatment but did not affect N74D HIV-1 trafficking. Alterations in HeLa trafficking under these conditions corresponded with HeLa infectivity data. These results again highlight the interplay of CPSF6 and CypA in capsid interaction. As it has not been possible to knockout CPSF6 or completely deplete CPSF6 from these cells, these data further suggest that the loss of CypA binding allows more CPSF6 binding to occur outside the nucleus, which affects trafficking and infectivity.

Although high-speed imaging of HIV-1 particles in CD4^+^ T cells was not possible, infection results after CPSF6 depletion and/or CsA treatment in primary PBMC largely mimicked those seen in HeLa cells. In addition, expression of CPSF6 and CypA are similar in CD4^+^ T cells and HeLa cells. However, CsA treatment significantly reduced the infectivity of WT HIV-1 as well as the CA mutants. Recent studies revealed that the loss of CypA binding, by either depletion, CsA treatment, or CA mutations, led to significant restriction of HIV-1 infection by human TRIM5α in CD4^+^ T cells (30, 52). Similarly, we show that trafficking of G89V HIV-1 complexes or WT HIV-1 in the presence of CsA was aberrant in MDM, which corresponded with reduced infectivity. As in CD4^+^ T cells, the loss of CypA binding to HIV-1 capsid in macrophages led to human TRIM5α restriction prior to completion of reverse transcription (29). Furthermore, N74D HIV-1 is restricted in macrophages and CD4^+^ T cells by TRIM34 in a TRIM5α-dependent and CPSF6-independent manner (52). Thus, CypA binding to HIV-1 capsid may be protective against multiple capsid-binding cellular factors. Further structural studies will be needed to understand the interplay of CypA, CPSF6, and TRIM5α/TRIM34 binding to WT and mutant HIV-1 capsids.

While some CA may be lost from the capsid in the cytoplasm, CA is required for docking at NPCs and for nuclear entry (11, 12, 53). Recent work from several groups has shown that capsid uncoating and reverse transcription are completed inside the nucleus in multiple cell types (14, 54, 55). However, some differences have been observed in different cell types. For example, imaging of HIV-1 capsids showed they were more stable in the cytoplasm of MDM than HeLa-derived cells (56). Similarly, most CA staining was lost from HIV-1 complexes in the nucleus of HeLa cells, while more viral complexes were CA positive in MDM (51, 56, 57) and colocalized with CPSF6 puncta (49, 55). Here, we show that CypA localized to both the cytoplasm and nucleus in MDM, whereas it was only expressed in the periphery of the cell in HeLa cells. Thus, HIV-1 capsid may be shielded from CPSF6 and other host factors by CypA for longer periods of time in MDM (Fig. 8). Also, little to no cytoplasmic CPSF6 puncta are detected in MDM. As we showed that CPSF6 binding to capsid was prevented by CypA binding and promoted capsid dissociation, CypA expression in the perinuclear region and nucleus of MDM could explain why more CA remains associated with the viral genome in these cells compared to HeLa cells. Our results contribute to the growing literature on the ability of HIV-1 capsid to bind multiple host cell factors in a highly orchestrated manner to promote viral infectivity, which differs depending on the specific cellular environment.

## MATERIALS AND METHODS

### Plasmids.

The replication-defective HIV-1 proviral plasmid pNLdE-luc has been described (21, 48). Viruses were pseudotyped with pL-VSV-G (58). N74D was also introduced similarly in a replication-competent proviral plasmid, pNL4-BAL (gift from Ned Landau). pVpr-mRuby3-IN was previously described (43), and a similar version, pVpr-tagRFP-IN was also used.

Lentiviral vectors were used to introduce tagged host proteins or shRNA to cells. Fluorescently tagged CPSF6 and related mutants were introduced into the pSICO vector using the BamHI and NotI sites. pSICO-GFP-TNPO3 was created using same sites with GFP-TNPO3 (gift from Ned Landau). pLenti-FAP-GPI was created using the BamHI and NotI sites to move the FAP-GPI sequence from pcDNA-IgKappa-myc-dL5-2XG4S-GPI (Addgene; catalog no. 153308) into pLenti-puro (gift from Ie-Ming Shih; Addgene; catalog no. 39481). A plasmid encoding CypA-DsRed (46) was a gift from Greg Melikyan. The lentiviral vector pHIVSIREN expressing CPSF6 shRNA (59) was a gift from Greg Towers. pLKO.1-puro-shNT (gift from Jacob Corn; Addgene; catalog no. 109012) was used as a scrambled shRNA control. The lentiviral packaging plasmid psPAX2 (gift from Didier Trono; Addgene; catalog no. 109012) and pCMV-VSV-G were used to produce viruses.

The *CPSF6* gene and the MBP tag were amplified by PCR and subcloned into the pcDNA3.1(+) mammalian expression vector (Thermo Fisher Scientific) using the NEBuilder HiFi assembly kit (New England Biolabs) after linearization with the restriction enzymes Eco RV and Xba I. The resulting insert, designated MBP-His_6_-CPSF6-588, has a leading Kozak sequence, an N-terminal MBP tag, followed by a hexahistidine tag (His_6_).

HIV-1 CA and CA-NC were previously described (60, 61). In brief, they were cloned from the cDNA of *Pr55^Gag^*, which was obtained from the NIH AIDS Research and Reference Reagent Program, Division of AIDS, NIAID, NIH. Briefly, CA and CA-NC regions were amplified and subcloned into pET21 (EMD Chemicals, Inc.) using the NdeI and XhoI sites. Proteins were expressed and purified as previously described for Gag (ΔMA_15–100_Δp6) (2, 62). CypA-DsRed-Exp2 (gift from Greg Melikyan) was cloned into the pET28 vector, resulting in an N-terminal His_6_-tagged protein.

### Cells.

HeLa and HEK 293T cell lines were cultured in Dulbecco’s modified Eagle medium (DMEM; Thermo Fisher Scientific) supplemented with 10% fetal bovine serum (FBS; Atlanta Biologicals), 100 U/ml penicillin, 100 μg/ml streptomycin, and 2 mM l-glutamine (PSG; Thermo Fisher Scientific) at 37°C and 5% CO_2_. Stable cell lines were made by transduction with lentiviruses expressing fluorescently tagged host proteins followed by fluorescence-activated cell sorting. GHOST-R3/X4/R5 lentiviral reporter cells (63) were cultured in DMEM supplemented 10% FBS, PSG, 100 μg/ml Geneticin G418 (Thermo Fisher Scientific), 0.5 μg/ml puromycin (Invitrogen), and 100 μg/ml hygromycin B (Invitrogen). SupT1 cells were cultured in RPMI 1640 medium (Thermo Fisher Scientific) supplemented with 10% FBS and PSG at 37°C and 5% CO_2_.

Human PBMCs were isolated from leukapheresis obtained from the Central Blood Bank (Pittsburgh, PA) using Ficoll-Paque Plus (GE Healthcare) density gradient centrifugation following the manufacturer’s instructions. PBMCs were cultured in RPMI 1640 medium supplemented with 10% FBS, PSG, and 20 U/ml recombinant interleukin-2 (IL-2; Thermo Fisher Scientific) at 37°C and 5% CO_2_. To expand T lymphocytes, PBMCs were stimulated with 50 U/ml IL-2 and 5 μg/ml phytohemagglutinin (PHA; Sigma-Aldrich) for 72 h prior to infection or transduction. CD14^+^ monocytes were isolated from PBMCs using human anti-CD14 magnetic beads with LS columns (Miltenyi Biotec). CD14^+^ monocytes were differentiated into MDM in RPMI 1640 medium supplemented with 10% FBS, PSG, and 50 ng/ml recombinant granulocyte-macrophage colony-stimulating factor (R&D Systems) for 7 days at 37°C and 5% CO_2_ prior to experimentation.

### Viruses.

Replication-defective HIV-1_NL4-3_-luciferase virus pseudotyped with VSV-G was produced by transfection of HEK 293T cells or HEK 293T cells stably expressing FAP-GPI with pNLdE-luc, pL-VSV-G, and pVpr-pcs-mRuby3-IN/pVpr-tagRFP-IN/CypA-DsRed at a weight ratio of 5:5:1. Replication-competent HIV-1 was produced by transfection of HEK 293T cells with the full-length proviral construct. Lentiviruses encoding fluorescent host proteins were produced by transfecting HEK 293T cells with lentiviral, packaging, and pCMV-VSV-G plasmids at a weight ratio of 4:3:1. Transfections were performed using Lipofectamine 2000 (Invitrogen). Viruses were filtered through a 0.45-μM filters, concentrated with Lenti-X (TaKaRa Bio) following the manufacturer’s protocol, and stored at −80°C. Viruses were quantified by p24 enzyme-linked immunosorbent assay (ELISA) (XpressBio) and the titer was determined on GHOST-R3/X4/R5 cells. FAP-GPI, mRuby3-IN, and CypA-DsRed labeled viruses were assessed for labeling efficiency by total internal reflection fluorescence (TIRF) imaging.

### HIV-1 infection assays.

HeLa cells and differentiated macrophages were seeded in 24-well plates overnight and then transduced with shRNA-encoding viruses. Next, 48 h postransduction, cells were infected with equal p24 amounts of luciferase reporter viruses for 48 h. Cells were lysed and assessed for luciferase production (Promega) with a 1450 MicroBeta TriLux microplate luminescence counter (PerkinElmer). CPSF6 KD efficiency was assessed by CPSF6 antibody staining (NBP1-85676). PHA-stimulated PBMCs were transduced with viruses encoding shRNA and selected with 2 μg/ml puromycin for 72 h. KD efficiency was measured by Western blot analysis. PBMCs were restimulated with PHA and challenged with luciferase reporter viruses for 72 h prior to luciferase measurement. For assays including treatment with CsA, cells were treated with CsA (10 μM) at the time of plating and remained in drug-containing medium throughout the assay.

Replication of HIV-1 in macrophages was performed in duplicate by infecting transduced macrophages with WT or N74D HIV-1_NL4-BAL_ at a multiplicity of infection of 0.1. Supernatant was collected and new medium was added every 2 days. Viral replication was quantified by p24 production in the supernatant by ELISA (XpressBio) at day 8.

### Fluorescence microscopy.

For fixed HeLa cell or macrophage imaging, cells were plated in MatTek dishes overnight. Synchronized infections with VSV-G pseudotyped HIV-1 were performed by incubation at 4°C for 10 min, followed by aspiration of medium, addition of cold fluorescently labeled HIV-1 (5 ng p24), and further incubation at 4°C for 15 min to allow virus attachment. Cells were then incubated at 37°C for 20 min, followed by washing with warm medium and incubation in fresh medium. At 1 h postinfection, cells were washed with phosphate-buffered saline (PBS), pH 7.4, and fixed with 2% paraformaldehyde (PFA). For SupT1 cell imaging, cells were infected in 6-well plates at room temperature with 10 ng p24 equivalent of virus per million cells for 30 min and then at 37°C for 3.5 h. Cells were centrifuged and washed twice with PBS and fixed with 2% PFA for 15 min. MatTek dishes were precoated with Cell-Tak (Corning) following the manufacturer’s instructions at least 2 h before seeding SupT1 cells. After permeabilization with 0.1% TritonX-100 for 15 min, fixed samples were blocked with serum matching the secondary antibody for 45 min. Primary antibodies were added to the fixed cells in protein binding buffer (PBB) (2% bovine serum albumin in PBS) for 1 h and washed with PBB. Secondary antibodies were added to the cells in PBB for 1 h. After washing with PBB and PBS, the cells were stained with Hoechst (1:2,000) and mounted with a coverslip using gelvetol.

A Nikon A1 confocal microscope was used to acquire three-dimensional (3D) stack images of fixed samples with a 100 × 1.49 NA oil-immersion objective. LU-NV laser launch (Nikon) was used to emit lasers at 405 nm, 488 nm, 561 nm, and 640 nm. Fields of view were randomly chosen by quick scanning in the Hoechst channel. The ND Acquisition option in NIS-Elements software (Nikon) was applied to collect 3D multichannel imaging (1,024 by 1,024 pixels) with 2× line averaging. Images of 488-nm and 561-nm channels were acquired by gallium arsenide phosphide (GaAsP) detectors (Nikon). 3D stacks were acquired with 0.15- to 0.5-μm step intervals to cover the entire cell volume (6 to 10 μm) with a motorized piezo Z stage (Nikon).

For live-cell HILO imaging, a Nikon Ti TIRF microscope with a 100 × 1.49 NA oil-immersion objective and a Photometrics Prime 95B sCMOS camera was used. In multicolor live-cell imaging experiments, a Flight Lakes Instrumentation high speed filter wheel was used. Synchronized infections in HeLa cells or macrophages were performed as described above. After shifting to 37°C for 20 min, cells were washed with prewarmed fresh medium—FluoroBrite medium (Thermo Fisher Scientific) for HeLa cells or RPMI 1640 medium for macrophages. After 1 h postinfection, the MatTek dish was loaded on the stage insert and maintained at 37°C (Tokai Hit stage chamber). Each image was acquired at least 1 frame per second (FPS) to track viruses for 10 min. For visualizing microtubules, 1 μM SiR-tubulin (Cytoskeleton) was added to the medium 30 min prior to imaging. For visualizing viral membranes, the MG-B-Tau FAP dye (64) was added to the virus at 500 nM for 10 min prior to addition to cells. RAM capture in Nikon Elements was used to achieve faster multicolor live-cell imaging (≥2 FPS).

### Imaging quantification and data analysis.

All imaging quantification was performed with General Analysis 3 in Nikon Elements (5.20.00 or above). Briefly, a cell nuclei binary mask was created using Hoechst signal to calculate the number of cells in each field of view. CPSF6 localization and quantification were determined by creating binary masks of CPSF6 within the cells. Cytoplasmic CPSF6 was determined by subtracting the CPSF6 binaries from ones colocalized with Hoechst (nucleus) signal. Mean intensity and volume were recorded for each binary. Virus localization was determined with the spot detection function to create binary masks for spots positive for mRuby3/tagRFP, FAP-GPI, CypA-DsRed, or p24 signals. Trafficking data of HIV-1 was determined by using the track function with the spot binaries in random and constant motion mode. Any tracks with less than 20 frames were excluded from the data analysis.

### Protein expression and purification.

The full-length CPSF6 protein was expressed in a suspension-adapted HEK293 cell line (Expi 293F; Thermo Fisher Scientific) by transfection of expression plasmid using ExpiFectamine 293 (Thermo Fisher Scientific) according to the manufacturer’s instructions. Following transfection, the cells were grown at 37°C by shaking at 125 rpm in 8% CO_2_ and 80% humidity for 2 days. The cells were harvested after 48 h of transfection by centrifugation at 100 × *g* for 10 min. The cell pellet was washed with cold PBS and flash-frozen and stored at −80°C.

The thawed cell pellet was resuspended in buffer A (50 mM HEPES-KOH [pH 8], 500 mM NaCl, and 5% glycerol, 2 mM dithiothreitol [DTT]) supplemented with detergents (1% Tween 20 and 0.3% NP-40) and DNase I (50 μg/ml; Sigma-Aldrich) in the presence of a cocktail of protease inhibitors (Roche). After 2 h of rotation at 4°C, the lysate was homogenized by 15 strokes in an ice-cold, tight-fitting Dounce homogenizer. The homogenate was then centrifuged at 21,000 × *g* at 4°C for 30 min. After centrifugation, the supernatant was collected and mixed with 1 ml of amylose agarose resin (New England Biolabs) preequilibrated with buffer A per 50 ml of cell homogenate. The mixture was incubated with rotation at 4°C for 2 h and then transferred to a column. The resin was washed with 50× resin volume of buffer A. To elute the recombinant protein, the resin was incubated with buffer A containing 100 mM maltose for 15 min at 4°C, and the flowthrough was collected as eluate. The eluate was applied to a Hi-Load Superdex 200 16/60 column (GE Healthcare) in a buffer A, and fractions containing target protein were collected and concentrated to 6 to 8 mg/ml using Amicon concentrators (Millipore, Billerica, MA, USA), flash-frozen with liquid nitrogen, and stored at −80°C.

His_6_-tagged CypA-DsRED-Exp2 was expressed in Escherichia coli Rosetta 2 (DE3) cells (EMD Millipore) with autoinduction medium at 18°C for 16 h. Protein was purified using 5 ml Ni-NTA column (GE Healthcare) and a HiLoad Superdex 200 16/60 size exclusion column (GE Healthcare) equilibrated with a buffer containing 25 mM sodium phosphate, pH 7.5, 150 mM NaCl, 1 mM dithiothreitol (DTT), 10% glycerol, and 0.02% sodium azide.

### SDS-PAGE and Western blot analysis.

For *in vitro* CPSF6 experiments, an equal volume of cell lysate and each fraction from the column were mixed with 4× NuPAGE lithium dodecyl sulfate (LDS) sample buffer (Thermo Fisher Scientific) supplemented with 10 mM DTT and loaded onto a 10% Bis-Tris NuPAGE gel (Thermo Fisher Scientific), alongside a protein molecular weight marker (BLUEstain protein ladder; Gold Biotechnology). Gels were run at 100 V for 15 min and then 150 V for 40 min in NuPAGE morpholineethanesulfonic acid (MES) SDS running buffer, and the proteins were subsequently transferred onto PVDF or nitrocellulose membranes using iBlot transfer stacks (Thermo Fisher Scientific). The membranes were blocked at ambient temperature for 1 h in bovine serum albumin (BSA) blocking buffers, followed by overnight incubation at 4°C with rabbit anti-maltose binding protein antibody (ab9084; Abcam) or rabbit anti-CPSF6 antibody (EPR12898; Abcam) and then a further hour with monoclonal anti-rabbit immunoglobulins-alkaline phosphatase antibody at ambient temperature. Between each antibody incubation, the membranes were washed three times with Tris-buffered saline (TBS) buffer containing 0.1% Tween 20, and finally, the membranes were developed with BCIP (5-bromo-4-chloro-3-indolylphosphate)/nitroblue tetrazolium (NBT) color development substrate (Promega) to enable visualization of protein bands (Promega, USA). Each experiment was performed at least three times.

For measurement of CPSF6 expression in cells, an equal number of transduced and puromycin-selected HeLa cells or PBMCs were lysed with RIPA buffer (Bio-Rad), mixed with sample buffer (Bio-Rad), and heated to 100°C for 5 min. Denatured cell lysate was run on precasted 4 to 15% Criterion Tris-HCl gels (Bio-Rad) at 150 V for 1.5 h. Proteins were transferred to nitrocellulose membranes using a semidry transfer apparatus (Thermo Fisher Scientific) at 160 mA for 1 h. The membranes were blocked with 5% milk in PBS containing 0.1% Tween 20 at room temperature for 20 min. The primary antibodies anti-CPSF6 (NBP1-85676) and anti-α-tubulin (T5168; Sigma-Aldrich) were used with secondary anti-mouse IgG or anti-rabbit IgG conjugated with horseradish peroxidase antibodies (A9917 and AP132P; Sigma-Aldrich). SuperSignal West Pico chemiluminescent substrate (Thermo Fisher Scientific) was used to visualize protein bands with Amersham hyperfilm (GE).

### Capsid binding assay.

Tubular assemblies of WT HIV-1 CA protein were prepared at 80 μM (2 mg/ml) in 1 M NaCl and 50 mM Tris-HCl (pH 8.0) buffer at 37°C for 1 h. N74D CA was dialyzed against 1 M NaCl and 50 mM Tris-HCl (pH 8.0) buffer at 4°C overnight at the concentration of 20 mg/ml. Before binding, the assembled mixture was diluted to 80 μM (2 mg/ml). For the binding assays, the binding buffer was the same as the stock buffer for MBP-CPSF6 proteins described above. Different concentrations of MBP-CPSF6 were added to preassembled CA tubes at a CA concentration of 64 μM. The reaction mixtures were incubated on a rocking platform at room temperature for 1 h with gentle mixing at 10-min intervals. Then, 5-μl samples were withdrawn from the reaction mixtures and immediately used for electron microscopy (EM) analysis. The remaining samples were pelleted at 21,000 × *g* for 30 min, and supernatants (s) and pellets (p, resuspended in the same volume) were mixed with 4× LDS loading buffer for gel analysis. Supernatant and pellet samples, without boiling, were loaded on 10% SDS-PAGE and stained with Coomassie blue. Each experiment was performed at least three times.

To determine the binding ratio of MBP-CPSF6:CA, SDS-PAGE gels were scanned using an Epson 4990 scanner. The integrated intensities of CA and MBP-CPSF6 protein bands were measured using the ImageJ 1.40 program (NIH). The molar ratios were calculated according to the formula (MBP-CPSF6 intensity/MBP-CPSF6 molecular weight)/(CA intensity/CA molecular weight) and calibrated using the input ratios as standards.

For binding of CypA and MBP-CPSF6 with HIV-1 capsid, 5 μM CypA-DsRed was added to 10 μM preassembled WT CA-NC tubes, and at the same time 15 μM competitive inhibitor CsA was added as a negative control. The reaction mixtures were incubated on a rocking platform at room temperature for 1 h with gentle mixing at 10-min intervals. Then, 5 μM MBP-CPSF6 P1 or P2 was added to the reaction and incubated on a rocking platform at room temperature for 1 h with gentle mixing at 10-min intervals. At the end of the incubation, the samples were pelleted as described above. Each experiment was performed at least three times. Nikon Elements 5.0 was used to quantify the binding ratio of CypA and MBP-CPSF6 with preassembled CA-NC tubes.

### TEM analysis.

The morphologies of different variants of CA assemblies and CA–MBP-CPSF6 complexes were characterized by TEM. Samples were stained with fresh 2% uranyl formate, deposited onto 400-mesh carbon-coated copper grids, and dried for 30 min. TEM images were acquired on a Tecnai T12 transmission electron microscope at 120 kV.

### Statistics.

Each virus infection experiment and associated imaging analysis was performed in at least two separate replicates. Compiled data were obtained from at least two independent experiments or three donors. Statistical significance was determined by two-sided unpaired Student’s *t* test using Prism (GraphPad). *P* values of <0.05 were considered statistically significant. ns, *P* > 0.05; *, *P* = 0.01 to 0.05; **, *P* = 0.01 to 0.001; ***, *P* = 0.001 to 0.0001; ****, *P* < 0.0001.
