# Supplementary figures and images for: Cytoplasmic CPSF6 Regulates HIV-1 Capsid Trafficking and Infection in a Cyclophilin A-Dependent Manner
Source: mBio. 2021 Mar 23;12(2):e03142-20. doi: 10.1128/mBio.03142-20 (PMC8092277; doi:10.1128/mBio.03142-20)

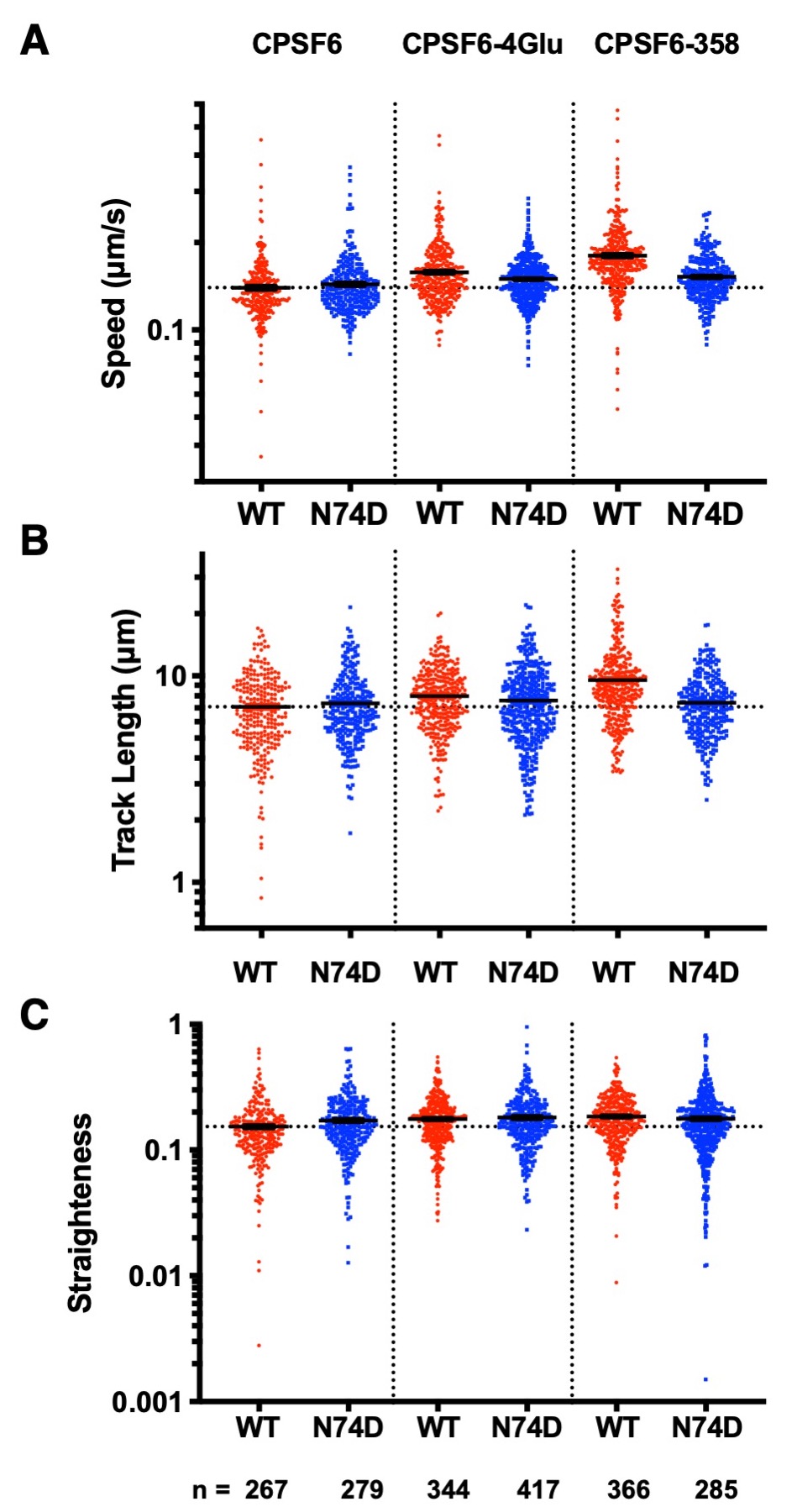

Supplement: FIG S1 [file mBio.03142-20-sf001.jpg]

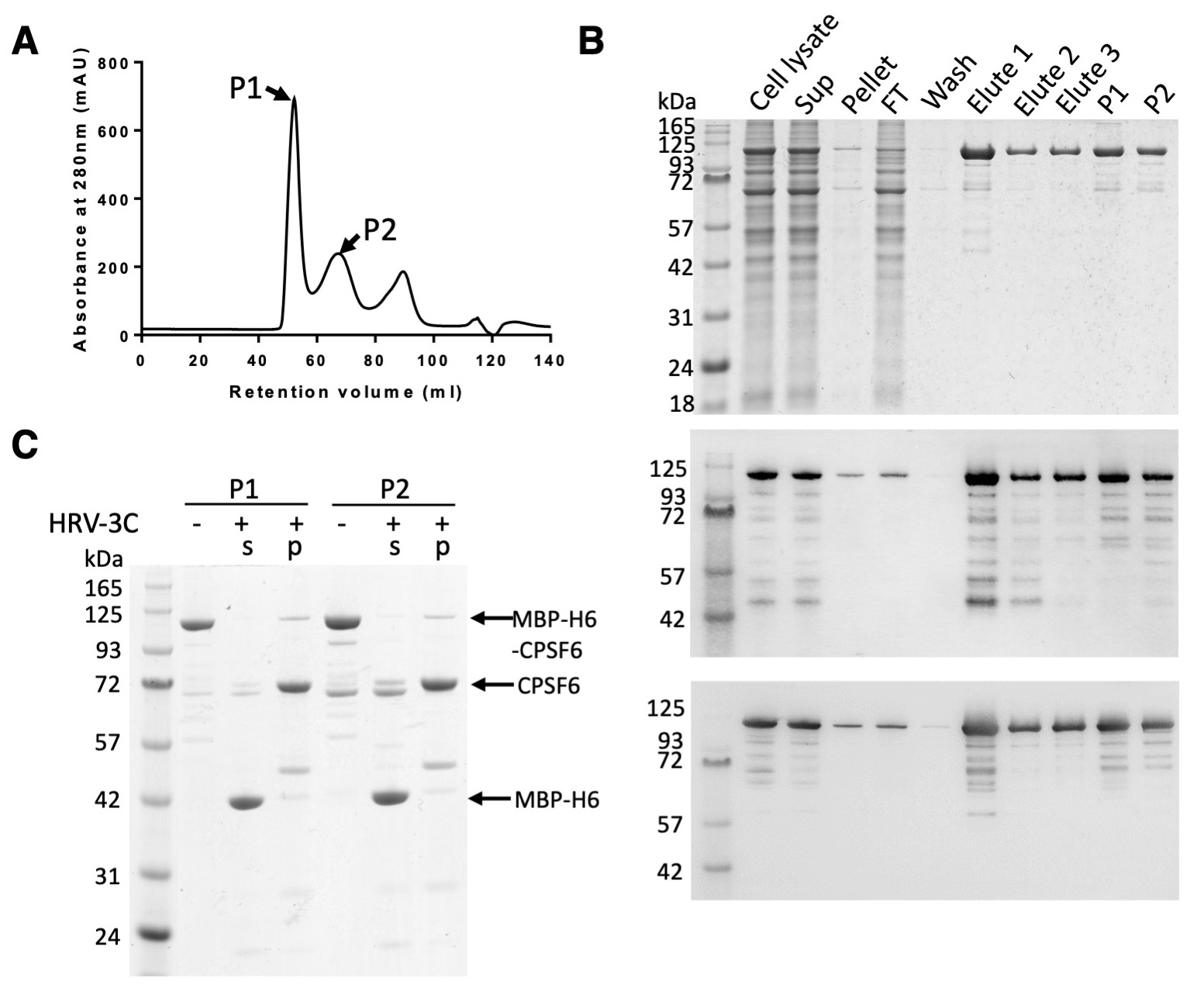

Supplement: FIG S2 [file mBio.03142-20-sf002.jpg]

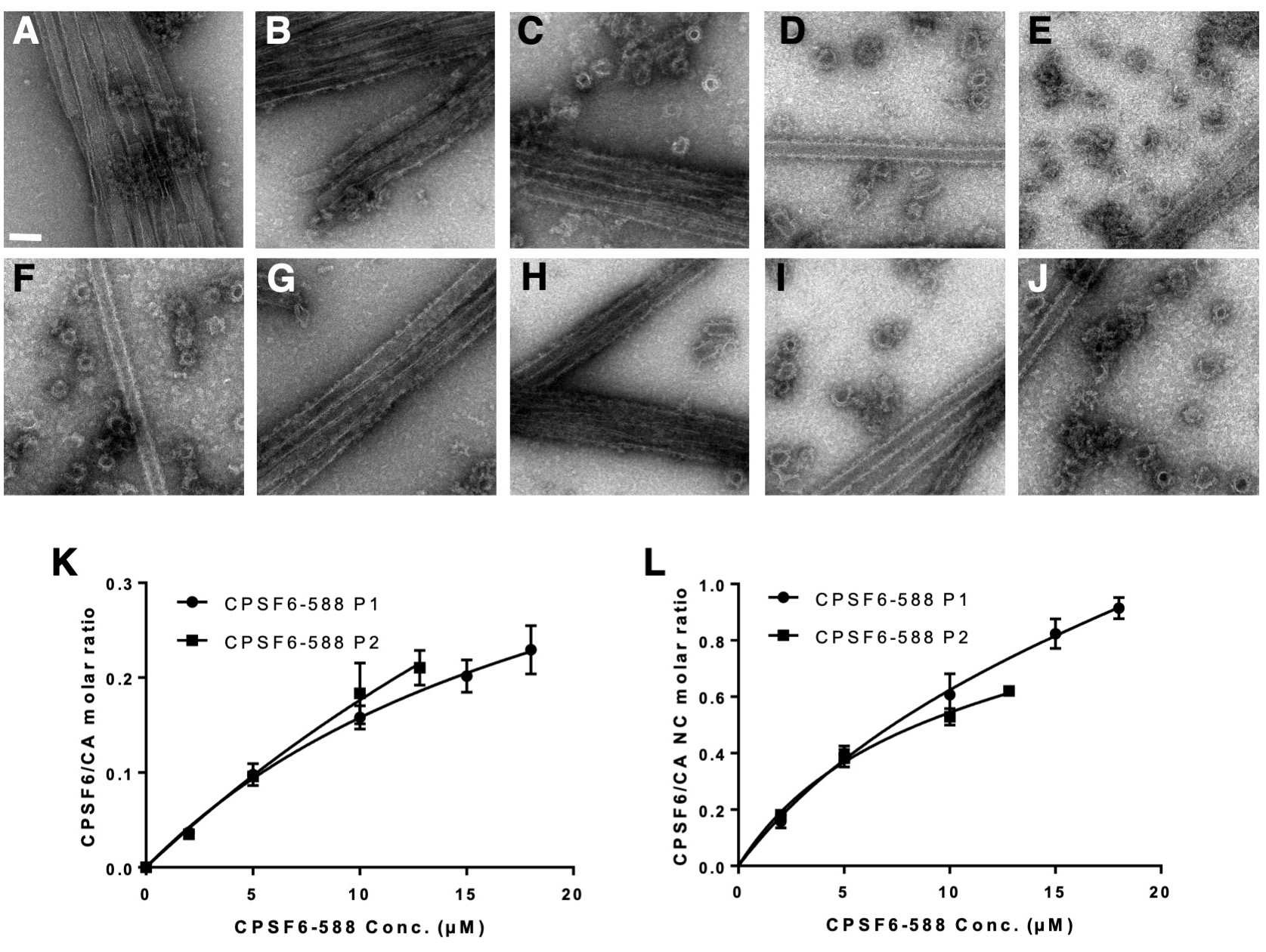

Supplement: FIG S3 [file mBio.03142-20-sf003.jpg]

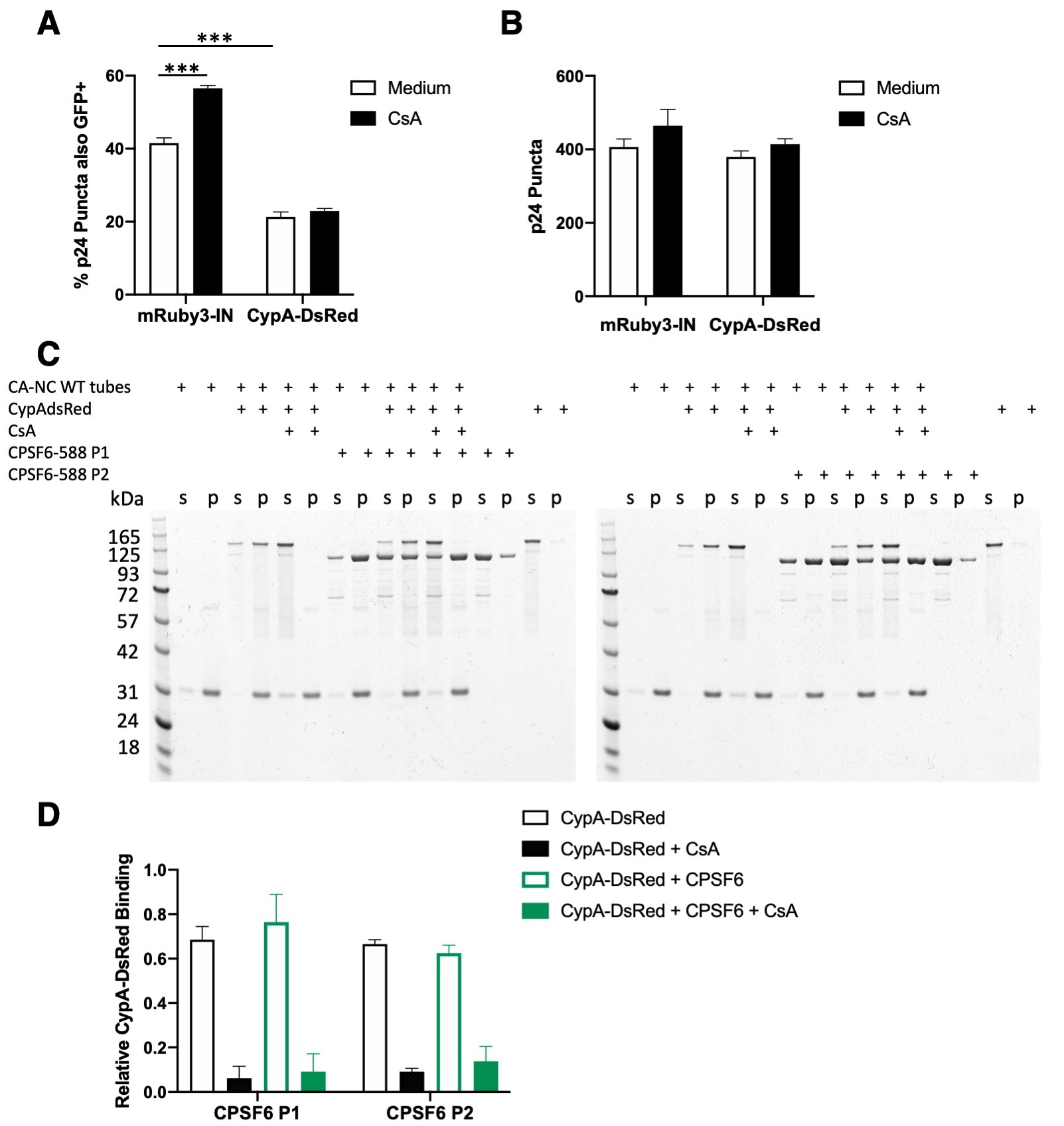

Supplement: FIG S4 [file mBio.03142-20-sf004.jpg]

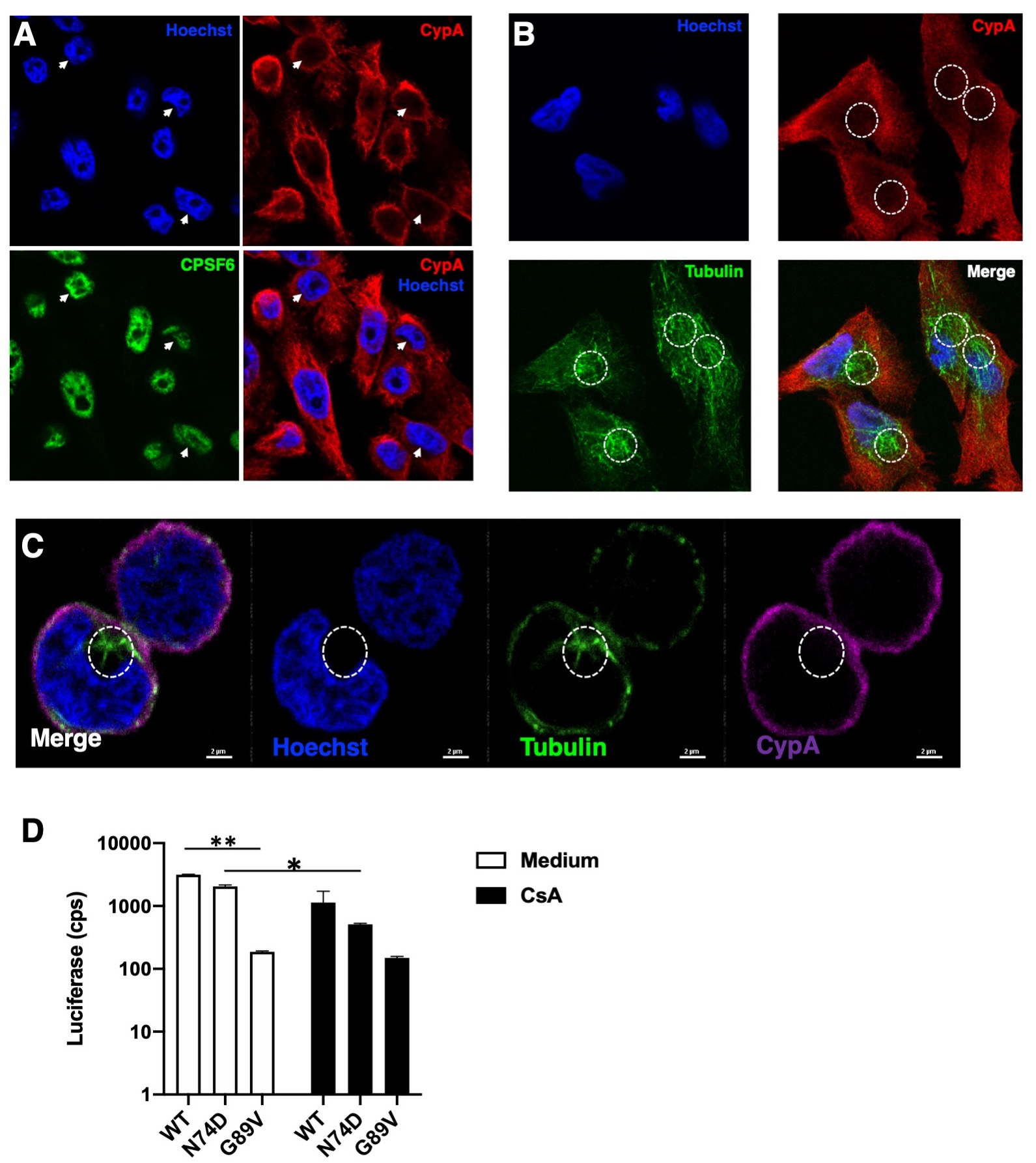

Supplement: FIG S5 [file mBio.03142-20-sf005.jpg]

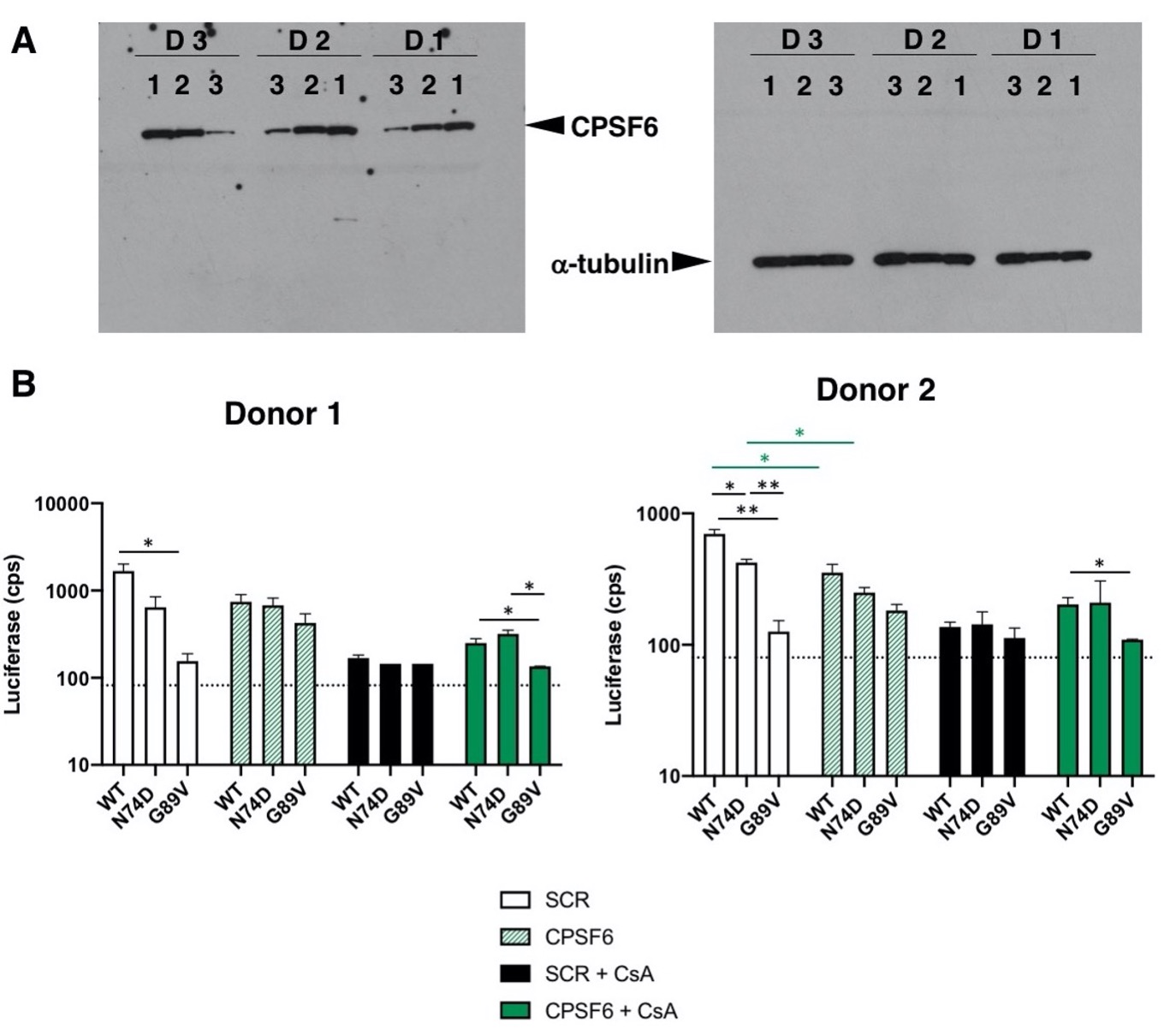

Supplement: FIG S6 [file mBio.03142-20-sf006.jpg]
